# Nanotopography Influences Host–Pathogen Quorum Sensing and Facilitates Selection of Bioactive Metabolites in Mesenchymal Stromal Cells and *Pseudomonas aeruginosa* Co-Cultures

**DOI:** 10.1021/acsami.4c09291

**Published:** 2024-08-08

**Authors:** Rosalia Cuahtecontzi Delint, Mohd I. Ishak, Penelope M. Tsimbouri, Vineetha Jayawarna, Karl V. E. Burgess, Gordon Ramage, Angela H. Nobbs, Laila Damiati, Manuel Salmeron-Sanchez, Bo Su, Matthew J. Dalby

**Affiliations:** † Centre for the Cellular Microenvironment, School of Molecular Biosciences, College of Medical, Veterinary and Life Sciences, Mazumdar-Shaw Advanced Research Centre, University of Glasgow, Glasgow G11 6EW, United Kingdom; ‡ Bristol Dental School Research Laboratories, Dorothy Hodgkin Building, University of Bristol, Bristol BS1 3NY, United Kingdom; § EdinOmics, University of Edinburgh, Max Born Crescent, Edinburgh EH9 3BF, United Kingdom; ∥ Safeguarding Health through Infection Prevention (SHIP) Research Group, Research Centre for Health, Glasgow Caledonian University, Glasgow G4 0BA, United Kingdom; ⊥ Department of Biological Sciences, College of Science, University of Jeddah, Jeddah 23218, Saudi Arabia

**Keywords:** Nanotopography, active coatings, antibacterial, quorum sensing molecules, metabolomics

## Abstract

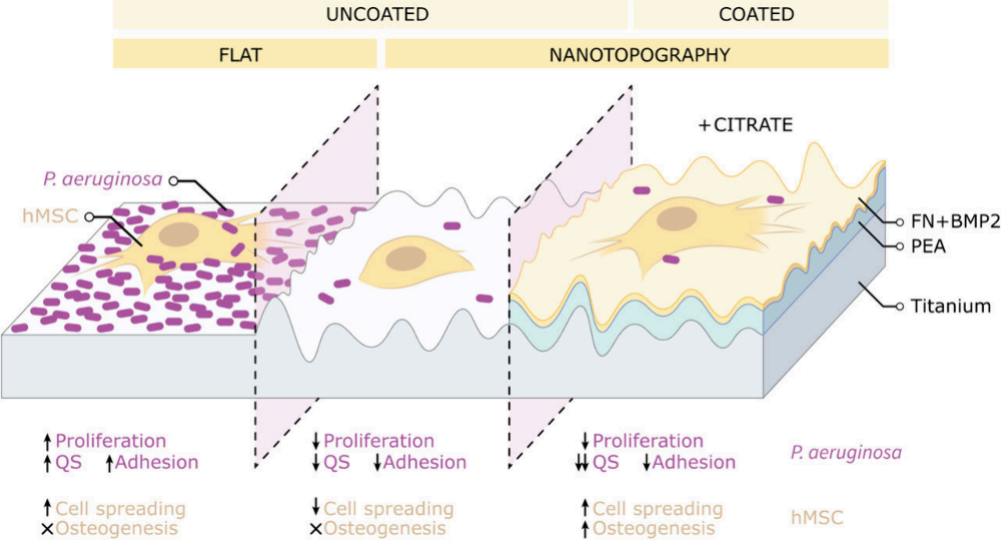

Orthopedic implant-related bacterial infections and resultant antibiotic-resistant biofilms hinder implant–tissue integration and failure. Biofilm quorum sensing (QS) communication determines the pathogen colonization success. However, it remains unclear how implant modifications and host cells are influenced by, or influence, QS. High aspect ratio nanotopographies have shown to reduce biofilm formation of *Pseudomonas aeruginosa*, a sepsis causing pathogen with well-defined QS molecules. Producing such nanotopographies in relevant orthopedic materials (i.e., titanium) allows for probing QS using mass spectrometry-based metabolomics. However, nanotopographies can reduce host cell adhesion and regeneration. Therefore, we developed a polymer (poly(ethyl acrylate), PEA) coating that organizes extracellular matrix proteins, promoting bioactivity to host cells such as human mesenchymal stromal cells (hMSCs), maintaining biofilm reduction. This allowed us to investigate how hMSCs, after winning the race for the surface against pathogenic cells, interact with the biofilm. Our approach revealed that nanotopographies reduced major virulence pathways, such as LasR. The enhanced hMSCs support provided by the coated nanotopographies was shown to suppress virulence pathways and biofilm formation. Finally, we selected bioactive metabolites and demonstrated that these could be used as adjuncts to the nanostructured surfaces to reduce biofilm formation and enhance hMSC activity. These surfaces make excellent models to study hMSC–pathogen interactions and could be envisaged for use in novel orthopedic implants.

## Introduction

Demand for total hip replacement (THR) and total knee replacement (TKR) implant surgeries is increasing worldwide due to the degeneration of bone among aging populations and those with active lifestyles,^1^ and while the success rate of these implants is initially high, many fail within 10 years.^2^ The leading causes of failure are aseptic loosening or bacterial infection on the implant surface.^3^*Pseudomonas aeruginosa* (*P. aeruginosa*), a biofilm-forming, sepsis-causing pathogen, accounts for up to 20% of Gram-negative implant infections after THR or TKR.^4^ Such biofilms pose significant challenges in healthcare settings, as they can be highly resistant to antibiotics. Therefore, developing strategies to inhibit bacterial attachment to the implant surface and disrupt biofilm formation is of importance, not only to improve the implant success but also to address the emergence of antibiotic resistance among pathogenic microorganisms.

Titanium (Ti) and its alloys are among the most widely used materials in joint replacement surgeries due to their similar properties to bone: good mechanical properties (high strength), low density, biocompatibility, and corrosive resistance.^5^ Additionally, these materials have been shown to significantly improve cell osseointegration *in vitro* and *in vivo.* Nevertheless, achieving optimal integration between the implant surface and surrounding tissue remains a challenge, yet is critical in preventing biofilm formation, since the race for the surface between regenerative host cells and pathogenic bacteria dictates the risk of infection.^6^

Previous work has shown that introducing high aspect ratio nanoscale topographies on Ti can confer antibacterial properties *in vivo* and *in vitro* while allowing mesenchymal stromal cell (human, hMSC) growth.^7,8^ Additionally, studies have shown that bacteria are less likely to adhere to high aspect ratio nanostructured Ti compared to smooth surfaces.^9−11^ First, this is because increased high aspect ratio features provide fewer contact points for bacterial adhesion as they rely on the physical interactions between their outer structures and the surfaces they colonize.^12^ Second, high aspect ratio nanoscale features can create an unfavorable environment for bacterial growth by altering their cell membrane in a way that can cause rupture and induce oxidative stress.^8,13,14^ Conversely, mammalian cells, display great tolerance to high aspect ratio nanotopographies^15^ due to the flexibility and strength of the cell membrane lipid bilayer.^9^ One drawback, however, is that while these high aspect ratio nanotopographies can maintain cell viability, they can reduce hMSC spreading,^9,15^ a key factor for osteodifferentiation.^16^

Considering this, one of the most effective ways to control cell–surface interactions involves bioconjugation of the surface with bioactive molecules such as peptides, cell adhesive proteins, extracellular matrix (ECM) proteins or growth factors.^17^ For instance, fibronectin (FN),^17^ collagen, bone morphogenetic protein 2 (BMP2), and peptides like the triamino acid sequence arginine-glycine-aspartate (RGD) have been used to influence hMSCs toward the osteoblast phenotype following attachment.^18−20^

We have previously explored the coating of high aspect ratio Ti nanotopographies using plasma polymerized poly(ethyl acrylate) (PEA). PEA can spontaneously organize FN into open nanonetworks; the open networks expose the RGD integrin binding site and the promiscuous growth factor binding site, the heparin binding region.^21^ The networks can then be used to promote hMSC adhesion and deliver BMP2 loaded onto open conformation FN in solid phase presentation to the cells.^21^ This synergizes integrin-related and BMP2 signaling and provides an effective growth factor signal at a lower dose due to slow internalization of the receptors when interacting with solid-phase BMP2.^21,22^

Following its placement, effective attachment of hMSCs to the surface of an implant will drive osseointegration,^23^ while attachment by bacterial cells will promote biofilm formation and subsequent infection. This underpins the race to the surface concept, with the outcome of competition between these two cell types for implant surface attachment translating into the likelihood of implant success. Nevertheless, how this race for the surface affects biofilm communication (via quorum sensing molecules, QSMs) and pathogen–host crosstalk is not well understood. It has been shown that surfaces that support hMSC attachment make them more resilient to virulence factor QSMs.^8^ QSMs can cause apoptosis and immune response regulation for the host, minimizing the host defense response and hence allowing sufficient bacteria to accumulate.^24,25^ Therefore, in this work, we focus on developing a metabolomics approach to understand changes in *P. aeruginosa* QSMs in response to hMSCs and high aspect ratio Ti nanotopographies. We explore the capacity for a PEA+FN+BMP2 coating on the Ti nanotopographies to tip the balance in favor of hMSC attachment. Specifically, we compare two Ti nanotopographies exhibiting different high aspect ratio nanotopographies to investigate the race to the surface in co-cultures of hMSCs and *P. aeruginosa*.

An improved, more mechanistic understanding of the race to the surface will provide new opportunities for the development of functional biomaterials for medical implants and devices that simultaneously reduce the risk of infection through their antibacterial properties, while improving osseointegration.

## Results and Discussion

Synthesis and characterization of 2D functional nanotopographies

**Figure 1:**
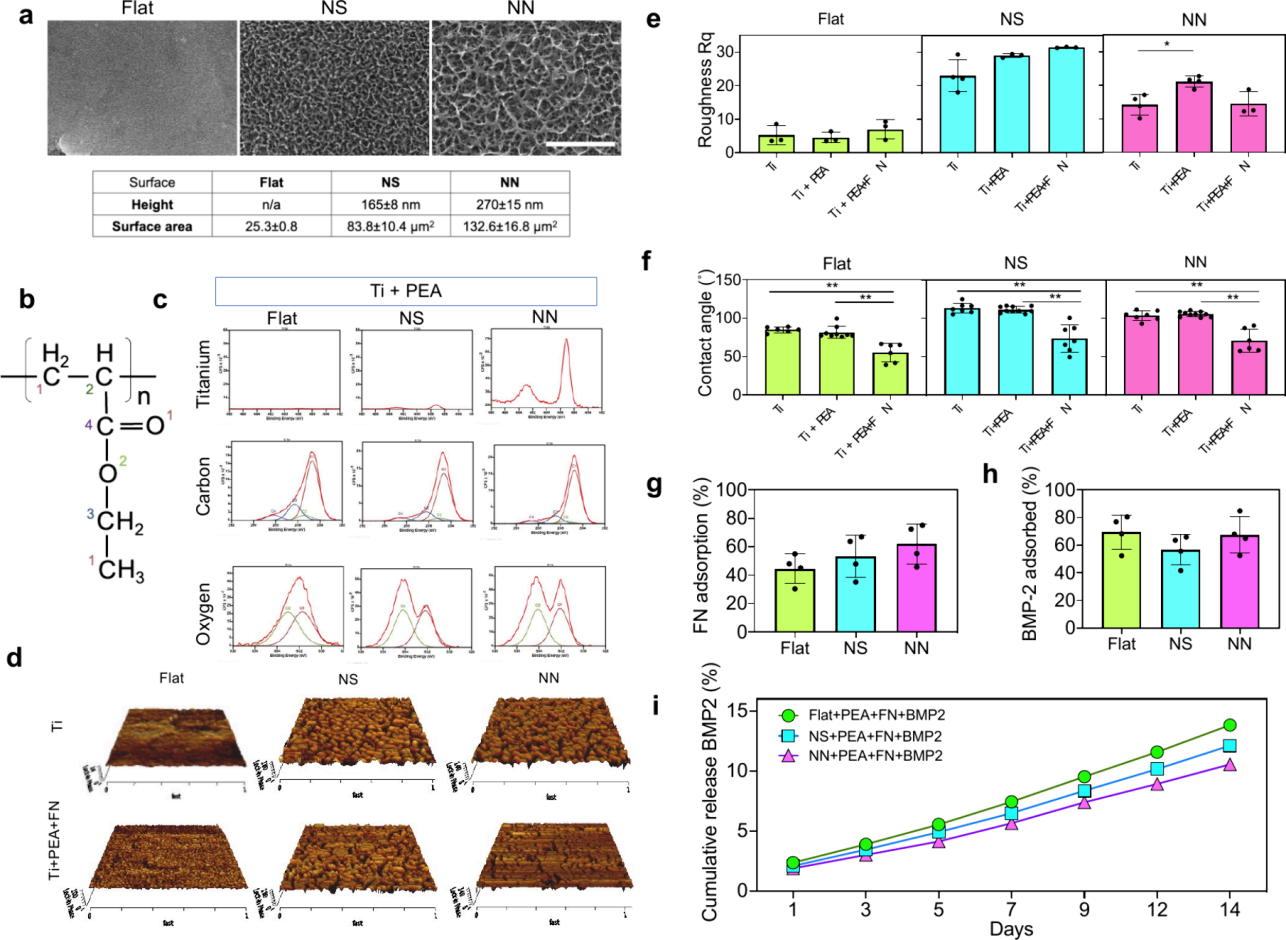
Characterization of functional active coatings on Ti nanotopographies. (a) Representative SEM images of flat control, nanospike (NS), and nanonetwork (NN) together with surface height and surface area measurements (table); scale bar 2 μm. (b) Chemical structure of PEA. (c) Titanium, carbon, and oxygen spectra of PEA coated flat, NS, and NN taken by X-ray photoelectron spectroscopy (XPS) surfaces. Each color corresponds to the number on the PEA chemical structure. (d) 1 × 1 μm AFM micrographs from the flat, NS, and NN surfaces before coating (top row) and after coating with PEA for 90 s at 100 W using plasma polymerization, followed by fibronectin (FN) for 1 h (bottom row). (e) Roughness (Rq) and (f) contact angle (3 μL sessile water drop) measurements for flat, NS, and NN with PEA coating and PEA+FN coating. ELISA was used to quantify the amount of (g) FN and (h) BMP2 adsorbed onto flat, NS, and NN samples after one h of coating. (i) The percentage of BMP2 released into solution after PEA+FN coating, quantified at days 1, 3, 5, 7, 9, 12, and 14. (e–h) Average represented as bars with individual values and standard deviation. Comparison of differences was tested using a Kruskal–Wallis test with a *p*-value <0.05 (*) considered significant, and <0.001 (**) highly significant. Together these results show that the PEA+FN+BMP2 coating could be used on Ti flat, NS, and NN nanotopographies to test with bacteria, hMSCs, and co-cultures.

Nanotopographies were generated by etching polished Ti discs with sodium hydroxide (NaOH) for two h to form nanospikes (NS), or 16 h for nanonetworks (NN). Scanning electron microscopy (SEM) analysis confirmed spike network formation on both NS and NN samples (Figure 1a). Atomic force microscopy (AFM) showed that feature height measured 165 ± 8 nm for NS, and 280 ± 15 nm for NN, which showed higher spikes and yielded a larger surface area (Figure 1a). All samples were then plasma polymerized with polyethyl acrylate (PEA) for 90 s at 100 W, which resulted in a 19.44 nm thick PEA coating over the nanotopographies (Figure S1). Similar coating depths have been previously shown using this approach.^8^

Energy dispersive X-ray spectroscopy (EDS) and X-ray photoelectron spectroscopy (XPS) were used to characterize the surface chemical composition of the samples and assess the presence of PEA on the flat, NS, and NN surfaces after coating (Figure S2, Figure 1b and c). EDS results indicated that PEA coated samples exhibited a higher amount of carbon than their uncoated counterparts. For XPS, the elements considered in our analysis were Ti, carbon, and oxygen. It was noted that the amount of visible Ti varied depending on the nanotopography; for instance, the absence of Ti maxima on the flat+PEA samples was associated with the surface being conformally covered by the layer of PEA.^8,26^ However, in the NS+PEA and NN+PEA sample spectra, the Ti peaks remained visible. For the NS+PEA samples, the Ti peak was very small, but on the larger NN+PEA features, the peaks were very prominent, likely showing that the coating, which was purposefully kept very thin so as not to change the high-aspect ratio topographies, was no longer conformal. As shown in Figure 1b, all four carbon moieties present in PEA, C–H (∼285.0 eV), C–COOR (∼285.4 eV), C O (∼286.6 eV), O–C=O (∼288.9 eV) were present on flat+PEA, NS+PEA, and NN+PEA. However, for the oxygen (O 1s) spectra, two prominent oxygen moieties from PEA, C=O (∼532.1 eV) and C–O–C (∼533.5 eV), were observed on all three coated nanotopographies. These results confirmed that the surfaces were coated with PEA.

It has been shown that adsorption of fibronectin (FN) onto PEA promotes the self-assembly of FN nanonetworks at the PEA interface.^21^ This exposes the integrin binding site in FN at FNIII_9–10_ and the growth factor binding domain at FNIII_12–14._21,^22^ In this study, FN was adsorbed onto the PEA-coated Ti surfaces to enhance the binding and controlled presentation of growth factor BMP2 and to promote synergistic integrin-mediated signaling.^21^

AFM was used to image and characterize the nanotopography on the Ti surfaces and to assess the development of FN nanonetworks before and after coating with PEA+FN (Figure 1d, and Figure S3). The nanonetworks were most apparent in the flat samples. The uncoated flat control exhibited no features, in contrast to flat+PEA+FN, which presented FN in a conformation indicating nanonetwork formation, as described previously.^21^ It was noted that plasma polymerized PEA forms very tight networks compared to spin coated PEA, for which networks have been easily identified.^8,21^ Similar behavior was assumed for the NS and NN. However, due to the scale of the nanotopographies compared to that of FN, it was not possible to observe them. It is important to note that the addition of the PEA and PEA+FN layers did not obscure the features of the nanotopographies (Figure 1d); this was the intention of applying a very thin (19.44 nm) PEA coating.

After the surface roughness (Rq) was measured, it was observed that the coating did not alter the roughness significantly. There was an initial increase in Rq with the PEA coating on the NN surface, but this reduced with the addition of FN, returning to the uncoated roughness level (Figure 1e). This observation provided further confidence that the PEA+FN coating did not obscure the Ti nanostructures. A similar trend occurred with peak to valley (Rt) measurements (Figure S4).

A degree of hydrophilicity is one of the requirements for biomaterials to promote cell adhesion and survival and yield a homogeneous initial cell seeding.^27^ The wettability of substrates, as measured by the contact angle, provides insight into material-cell interactions. A contact angle smaller than 90° is considered hydrophilic, while angles larger than 90° are considered hydrophobic. Sessile drop contact angle was measured on the uncoated flat, NS, and NN, and after PEA coating (flat+PEA, NS+PEA, and NN+PEA) and FN coatings (flat+PEA+FN, NS+PEA+FN, and NN+PEA+FN). In all cases, the uncoated controls and PEA coated nanotopographies exhibited hydrophobic behavior (flat = 85 ± 3°, NS = 113 ± 6° and NN = 103 ± 6°), with the nanostructures for NS and NN increasing hydrophobicity compared to flat surfaces (Figure 1f and Figure S5). Coating with PEA, a hydrophobic polymer,^21^ did not significantly change the hydrophobicity. However, addition of FN to the PEA caused the surfaces to become hydrophilic (flat = 55 ± 12°, NS = 74 ± 17°, and NN = 71 ± 14°).

The amount of FN deposited on the different Ti nanotopography surfaces was quantified using an enzyme linked immunosorbent assay (ELISA) after one h of coating. Data showed that the amount of FN increased on flat+PEA, NS+PEA, and NN+PEA coated surfaces, potentially reflecting increasing surface areas (Figure 1g). ELISA for BMP2 after PEA+FN coating showed no trend, with around 60% of BMP2 added to the surfaces remaining absorbed (Figure 1h). BMP2 remained adsorbed on the surfaces with less than 15% being released in solution over the course of 14 days (Figure 1i). Together, these findings suggested that a layer of PEA+FN+BMP2 could be used on the Ti flat, NS and NN surfaces and evaluated for its effects on surface interactions with bacteria and hMSCs independently and in co-culture. From here on, the PEA+FN+BMP2 coating (c) on flat, NS, and NN will be referred to as cFlat, cNS, and cNN, whereas the uncoated (u) Ti controls will be named as uFlat, uNS, and uNN for clarity.

### Ti Nanotopography Antibacterial Properties

Following attachment to an implant surface, bacteria become enclosed within a polymeric matrix as the biofilm develops.^28^ This layer protects bacteria against environmental threats, including antibiotics, in contrast to when they remain unattached or in planktonic phase. Inhibiting biofilm formation is, therefore, crucial in combatting implant infections.^28^ To evaluate the ability of the Ti surfaces to inhibit bacterial biofilm formation, *P. aeruginosa* was chosen as it accounts for up to 20% of Gram-negative implant infections following THR or TKR surgeries.^29^ This strain spontaneously develops biofilms and has a well understood quorum sensing (QS) system.^30,31^

As an opportunistic pathogen, *P. aeruginosa* exhibits a rapid population doubling time, ranging from 30 min to 2 h in a laboratory setting using a rich culture medium.^32^ We initially used a *P. aeruginosa* monoculture to specifically study bacteria-surface interactions and to optimize conditions for co-culture, where bacterial numbers needed to be controlled to allow assessment of the slower hMSC response (days) alongside a slowed bacterial response. First, a titration of penicillin/streptomycin (P/S) antibiotics versus colony forming units (CFU) of *P. aeruginosa* was conducted. Cultures ranging from 0.2% to 1% v/v P/S and 10^2^ to 10^6^*P. aeruginosa* CFU were set up in low serum (1%) cell media and viability was measured after 24 h (Figure S6a and S6b). These same cultures were then serially diluted, and agar plates were set up after a further 24 h incubation period (Figure S6c and S6d). A combination of 0.3% P/S and 10^3^ CFU was chosen for all subsequent experiments as these bacterial cultures remained viable, but population growth was controlled. These conditions were used for all experiments whether monoculture or co-culture.

To test the bactericidal activity of flat, NS, and NN, *P. aeruginosa* culture consisting of 10^3^ CFU were incubated for 24 h on uncoated (uFlat, uNS, and uNN) and PEA+FN+BMP2 coated flat, NS and NN (cFlat, cNS, and cNN) surfaces and visualized by SEM, following live/dead staining. Effects on QS signaling were also evaluated.

**Figure 2:**
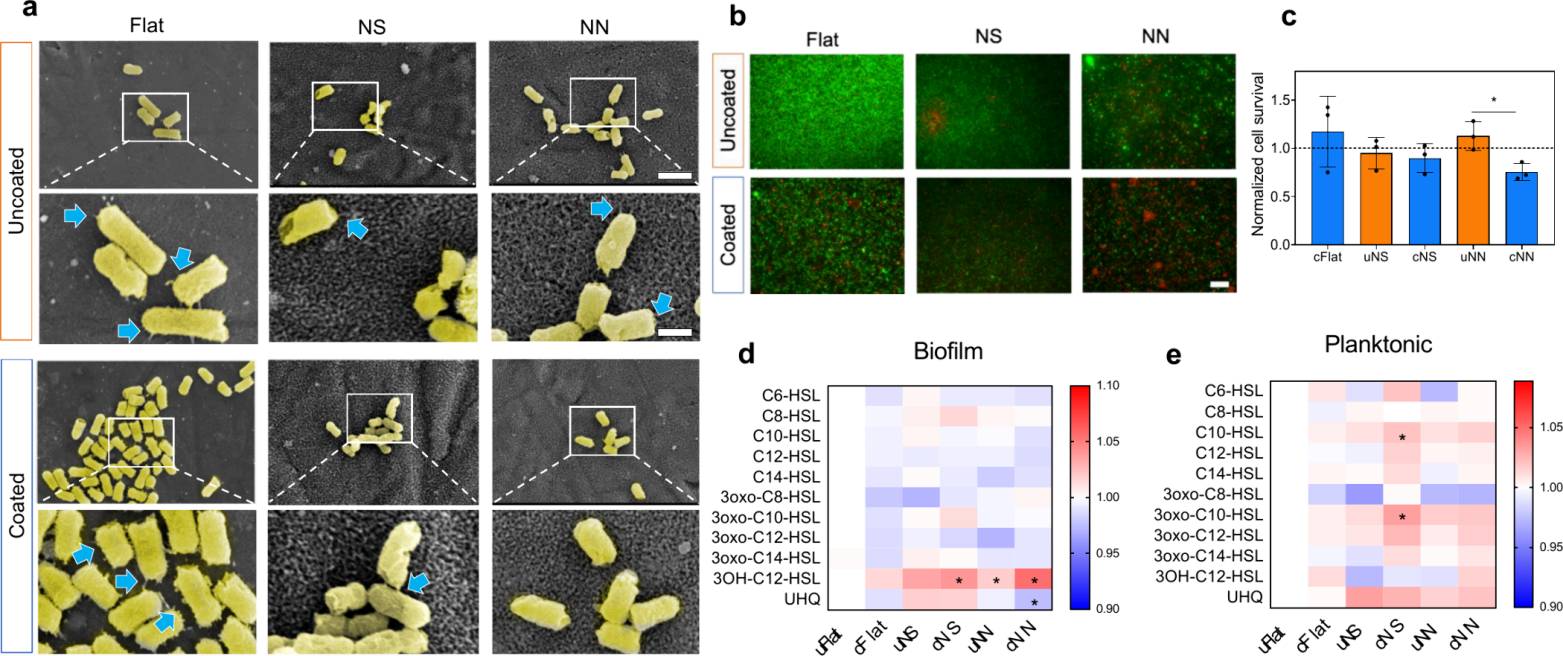
Effect of nanotopographies on*P. aeruginosa*. Bacteria were incubated on flat, NS, or NN coated or uncoated surfaces for 24 h. (a) The samples were fixed and visualized by SEM to assess morphology; scale bar, 2 μm (top panel) and 600 nm (bottom); blue arrows indicate cell surface appendages. (b) Live/dead staining indicates viable bacteria (green) or dead bacteria (red); scale bar, 50 μm. (c) The levels of cell survival quantified using Fiji, normalized to uFlat represented as bars with individual values and standard deviation. Statistical significance between conditions was tested using Kruskal–Wallis test with a *p*-value <0.05 (*) considered significant, and <0.001 (**) highly significant. Metabolites were isolated from the (d) biofilm and (e) planktonic bacteria, then submitted to triple quadrupole mass spectrometry to determine levels of QSM. Average expression is represented as a heatmap, where red-colored bars represent upregulation and blue downregulation compared to uFlat. Statistical significance between conditions was tested using a two-tailed unpaired homoscedastic *t* test with a *p*-value <0.05 (*) considered significant. These surfaces, particularly cNN, can inhibit bacteria biofilm formation and reduce QSM in *P. aeruginosa*.

First, we looked at cell surface appendages, as structures such as pili and nanotubes are known to influence the dynamics of *P. aeruginosa* attachment to surfaces including nanotopographies,^33^ and biofilm formation. Furthermore, pili are implicated in the transcriptional control of virulence factors and QS pathways.^34^ On the flat surfaces, whether uncoated or coated, bacteria were anchored via cell surface appendages, and cells exhibited an increased length compared to those on the nanotopographies (Figure 2a). Both uncoated and coated nanotopographies supported *P. aeruginosa* attachment, but cells appeared shorter and fewer cell surface appendages were visible, especially for bacteria on the cNN surface.^33^

Quantification of bacterial viability on the surfaces based on live/dead staining revealed that only the cNN surface showed a significant reduction in the bacterial viability (Figure 2b and c). The quantification of total attached bacteria can be found in Figure S7. Levels of adenosine triphosphate (ATP) were also used as a complementary measure of the bacterial viability. These data indicated a reduction in *P. aeruginosa* metabolic activity on both uncoated and coated nanotopographies and again, a significant difference was seen for bacteria on the cNN surface compared to the uNN surface (Figure S8).

The observed loss of cell surface appendages and decrease in viability seen for bacteria incubated on the nanostructured surfaces compared to the flat surfaces correlates with evidence that larger nanostructures exhibit greater antibacterial effects as they cause greater deformation of the bacterial cell envelope and thus elicit a stronger stress response.^35^ Indeed, high aspect ratio topographies that have a greater number of contact points inflict more damage to the cell envelope by applying pressure.^36^ Importantly, we show here that the effects of the nanotopographies were not masked by bioactive coatings such as PEA+FN+BMP2.^8,37^

We next wanted to understand if the changes seen in bacterial adhesion and morphology corresponded with changes in *P. aeruginosa* quorum sensing molecule (QSM) expression, since QSM release allows bacteria to sense their environment, regulate gene expression and communicate, and is an important factor in biofilm development.^38^ Gram-negative bacteria such as *P. aeruginosa* use acylated homoserine lactone signaling molecules (HSLs) as QSMs, which depend on regulatory circuits for the encoded transcriptional regulator LasR^39^ as well as the quinolone system.^40^

*P. aeruginosa* produces 3-oxo-C10-HSL (3-oxo-decanoyl-L-HSL), 3-oxo-C12-HSL (3-oxododecanoyl-L-HSL) and 3-oxo-C14-HSL (3-oxo-tetradecanoyl-L-HSL), which autoinduce the LasR system. Once activated, LasR works hierarchically with another transcriptional regulator, RhlR, to upregulate toxin, enzyme, and pili production.^41,42^

Quinolines also act as QSMs in *P. aeruginosa*. Alkylquinolones, such as 2-undecyl-4-hydroxyquinoline (UHQ), that were investigated here, act as autoinducers, accumulating in the bacterial cell until a signal threshold is reached and then activating a number of virulence-related genes. Furthermore, they have activity against competitor bacteria.^43,44^

The production of QSMs by *P. aeruginosa* was evaluated after 24 h incubation on uncoated and coated flat, NS and NN surfaces. For these studies, we developed a 29 compound QSM standard library based on *P. aeruginosa* literature^39^ (Table S1), which enabled the accurate and quantitative detection of each QSM through triple quadrupole mass spectrometry. Biofilm-forming (sessile) and suspension-based (planktonic) bacteria were analyzed separately.^45^ Mass spectrometry on both bacterial populations detected 11 of the QSMs in the library (Figure 2d and e; and Table S2). Figure 2d shows the QSMs that were identified as present in the bacterial samples and illustrates a QSM depletion pattern for sessile *P. aeruginosa* in the biofilm on the nanotopographies and on coated samples compared to uFlat; including reductions in 3-oxo-C10-HSL and 3-oxo-C14-HSL. By contrast, 3-oxo-C12-HSL was upregulated and statistically increased compared to uFlat on cNS and cNN. Interestingly, UHQ was elevated for bacteria cultured on NS, but significantly down regulated for bacteria cultured on NN surfaces. For planktonic cells (Figure 2e), a more general pattern of QSM upregulation was observed; this included for 3-oxo-C10-HSL, 3-oxo-C12-HSL, 3-oxo-C14-HSL and UHQ. The specific functions of each QSM in *P. aeruginosa* have not yet been elucidated; however, it is known that, as a whole, they contribute to the production of virulence factors and subsequent biofilm formation. The possible implications of these QSM can be found in Table S3.

Together these data imply that the biofilm resident *P. aeruginosa* population were repressed by the coating and the nanotopographies, particularly NN. The planktonic population, which are more vulnerable to antibiotics,^46^ were more active. Both the LasR and quinoline systems are implicated, but it is interesting to note that the RhlR regulating QSM, C4-HSL (butanoyl-L-HSL) was not detected. Activation of RhlR is essential in *P. aeruginosa* pathogenesis, but activation of LasR without activation of RhlR has been implicated in increased bacterial growth but without tissue damage.^47^ In contrast, when only the RhlR system was present, tissue damage has been observed and linked to the presence of rhamnolipids.^47^ Rhamnolipids are biosurfactants and a type of virulence factor produced by *P. aeruginosa* and are responsible for biofilm formation and tight-junction infiltration particularly with epithelial cells.^47^

### Coated Nanotopographies Enabled hMSC Adhesion

**Figure 3:**
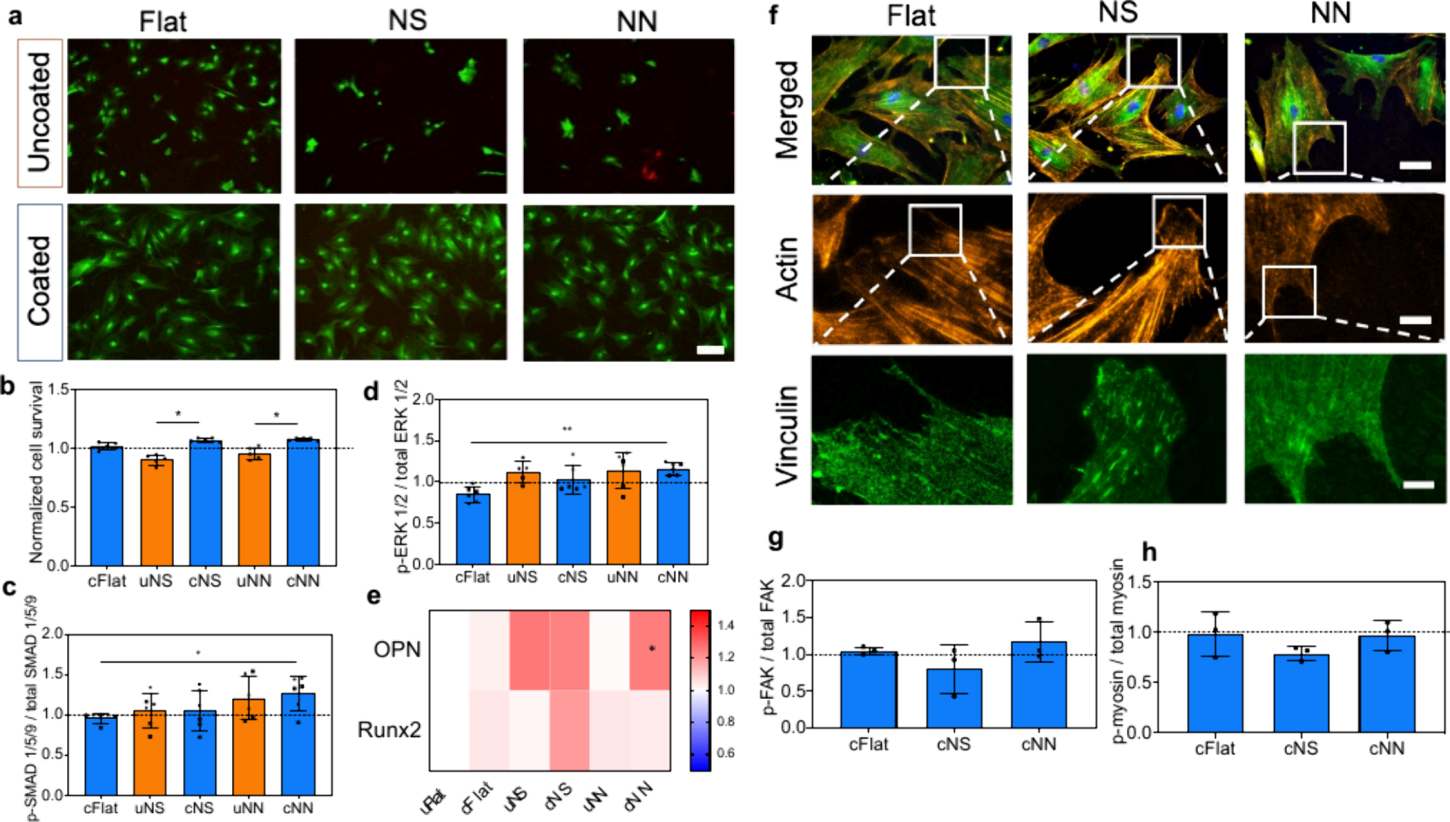
Effect of topographies on hMSCs. hMSCs were incubated on flat, NS, and NN topographies coated or uncoated using co-culture media for 24 h under a 5% CO_2_ atmosphere at 37 °C. (a) A live/dead assay was performed showing living (green) and dead hMSCs (red), scale bar = 50 μm. (b) Quantified using Fiji. ICW data comparing variations in protein expression of (c) p-ERK 1/2 and (d) p-SMAD 1/5/9 relative to their total proteins in hMSCs seeded on cFlat, cNS, cNN or uFlat, uNS, and uNN nanotopographies and cells on uFlat were taken as a control (black dotted line). (e) Protein expression of differentiation markers on hMSCs seeded for 14 days using co-culture media, average expression is represented in a heatmap, where red-colored bars represent upregulation and blue downregulation compared to uFlat control. Comparison of differences was tested using a Mann–Whitney test with a *p*-value <0.05 (*) considered significant. (f) Cells bound on Ti surfaces containing cells were stained for actin (orange) and vinculin (green) after 24 h incubation; scale bar is from top to bottom 100, 30, and 10 μm. (g) The expression of p-FAK was evaluated after 24 h of seeding using ICW. (h) p-Myosin was evaluated after 24 h of seeding using ICW. Average represented as bars with individual values and standard deviation (b-c and g-h). Comparison of differences was tested using a Kruskal–Wallis test with a *p*-value <0.05 (*) considered significant, and <0.001 (**) highly significant. Together, the data show that the nanotopographies lower hMSC adhesion and adhesion-related signaling, but the pPEA+FN+BMP2 coating allows better adhesion and thus osteogenic phenotype.

Stro-1^+^ enriched hMSCs were used to assess the in vitro cell-Ti interaction on flat, NS, and NN samples coated with PEA+FN+BMP2 (cFlat, cNS, cNN) and equivalent uncoated controls (uFlat, uNS, uNN), in monoculture. First, the viability of the hMSCs was measured 24 h after initial cell seeding onto the surfaces using live/dead stain. It was observed that hMSCs on all the uncoated samples were poorly spread and this became more pronounced as nanotopography was introduced (NS) and the aspect-ratio increased (NN), with dead cells also becoming visible (Figure 3a). In contrast, hMSCs on all coated surfaces were spread well and viable (Figure 3a). Further, the cell area and perimeters significantly decreased on the uncoated nanotopographies (NS and NN) compared with their coated counterparts (Figures S9 and S10). Quantification of viability and microscopy revealed that the coatings recovered cell survival on the nanotopographies (Figure 3b).

The ability of the hMSCs to form focal adhesions on the Ti surfaces was evaluated 24 h after seeding. Fluorescence microscopy using actin to visualize cytoskeletal microfilaments and vinculin to observe mature focal adhesions was performed on both uncoated control surfaces (Figure S11) and the coated surfaces (Figure 3f). The uncoated nanotopographies supported hMSCs but the cells formed very few discernible focal adhesions and had poorly organized stress fibers. By contrast, all of the coated surfaces supported hMSCs with well-developed adhesions and well-organized stress fibers (Figure 3f). Using in-cell western (ICW) for active focal adhesion kinase (p-FAK) versus total FAK, and active p-myosin versus total myosin, coated nanotopographies were found to stimulate equivalent levels of adhesion, activation, and cytoskeletal contraction to the flat controls (Figure 3g and 3h, and Figures S12 and S13). FAK associates with growing focal adhesions and forms a main signaling component of adhesions, influencing contraction through activating actin/myosin interactions and differentiation through influence of biochemical signaling hubs such as extracellular signal-related kinase 1/2 (ERK 1/2).^16^ That hMSCs could adhere normally to the coated nanotopographies and generate cytoskeleton-derived intracellular tension is important, since intracellular tension is implicated in hMSC osteogenic differentiation.^48,49^

As we use FN to both initiate adhesion and deliver BMP2, we used ICW to investigate ERK 1/2 as a downstream regulator of cell adhesion and small mothers against decapentaplegic (SMAD 1/5/9), implicated in the BMP2 receptor (BMPRIa) translocating to the nucleus via BMP2 canonical signaling. Both can result in activation of the master osteogenic transcriptional regulator, runt-related transcription factor 2 (Runx2).^50^

Short-term signaling from p-SMAD 1/5/9, and p-ERK 1/2 was evaluated after 24 h of the cells being seeded on uncoated control and coated Ti surfaces. Both adhesion-based (ERK 1/2) and BMP2-based (SMAD 1/5/9) signaling were seen to be equally stimulated on the coated nanotopographies compared to cFlat controls. It is notable that both SMAD 1/5/9 and ERK 1/2 were significantly higher on cNN compared to uFlat control (Figure 3c and 3d and Figures S14 and S15).

ICW was next used to screen for osteogenic markers after 14 days in co-culture media to assess the differentiation potential of the nanotopographies. hMSCs were screened for expression of Runx2 and osteopontin (OPN) (Figure 3e and Figures S16 and S17). Expression levels for both proteins were elevated in cells on all surfaces compared with the uFlat control surface. Increased expression trends were most notable for Runx2 and particularly OPN on the cNS and cNN samples.

Taken together, these data showed increased spreading and survival by hMSCs when the high aspect ratio nanotopographies had the PEA+FN+BMP2 coating, while poor hMSC spreading and cell death were seen for the uncoated nanotopographies. This is essential as adhesion is a key first step in hMSC function.^8,27^ Additionally, in the presence of the coating, hMSCs exhibited similar adhesion/growth factor expression and phenotypic characteristics when exposed to the nanostructures, as seen for hMSCs on the flat Ti. This is important, as effective implant surfaces must be nondetrimental (or even beneficial) to hMSCs while preventing bacterial biofilm formation.

### Nanotopographies and hMSCs Exert Cooperative Antibacterial Effects in Co-Cultures with *P. aeruginosa*

At this stage, cNN was seen to be the most effective surface against *P. aeruginosa* while maintaining hMSC adhesion and differentiation potential and, therefore, formed the focus for hMSC–*P. aeruginosa* co-culture experiments.

**Figure 4:**
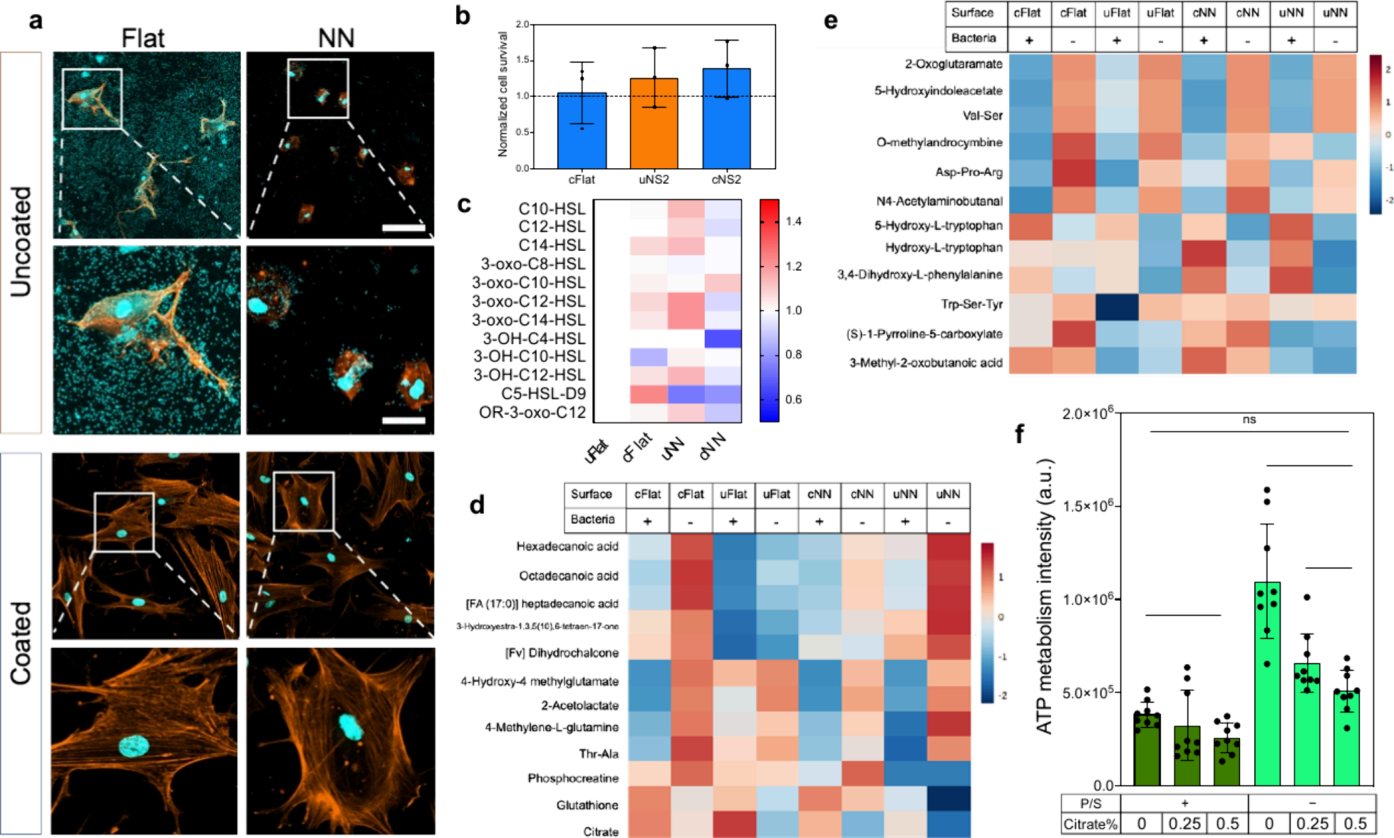
Effect of coating and nanotopography on hMSC–*P. aeruginosa* co-culture. hMSCs and *P. aeruginosa* were incubated for 24 h in co-culture on cFlat, cNN, uFlat, or uNN surfaces. (a) DAPI was used to observe hMSC nuclei and bacteria (blue), and actin (orange) was used to observe the cell morphology of hMSCs; scale bar, 100 μm (top row), 30 μm (bottom row). (b) A live/dead assay was performed and quantified using Fiji with the data normalized to uFlat. (c) The QSMs produced by *P. aeruginosa* were measured by high-resolution mass spectrometry. (d) The metabolites from hMSCs, and (e) the secretome were extracted, measured using liquid chromatography mass spectrometry, and analyzed using Metaboanalyst 5.0. (f) *P. aeruginosa* was seeded onto the surfaces overnight and the next day, incubated in media containing 0, 0.25, or 0.5% (w/v) citrate for 24 h under a 5% CO_2_ atmosphere at 37 °C, then, a BacTiter-Glo assay was performed, and the luminescence measured. Average represented as bars with individual values and standard deviation (b and f). An unpaired *t* test was performed in c, and a Kruskal–Wallis test with a *p*-value <0.05 (*) considered significant, and <0.001 (**) highly significant for f. NN acts in synergy with c to inhibit biofilm formation in co-culture and enhance hMSC viability, and that citrate cycle is upregulated by hMSCs in culture.

hMSCs were seeded overnight on uFlat, cFlat, uNN, and cNN, and after 24 h, 10^3^ CFU of *P. aeruginosa* were added and incubated in co-culture overnight. Fluorescence microscopy with labeling for actin microfilaments (Figure 4a), SEM (Figure S18) and live/dead staining (Figure S19) showed a greater degree of cell spreading for hMSCs on both surfaces in the presence of the PEA+FN+BMP2 coating. Despite dead cells being visible on the uNN (Figure S19), hMSC viability was high on all surfaces (Figure 4b). Cell area and perimeter were also higher when all cells on cNN were compared versus uNN (Figure S20 and S21).

The antimicrobial properties of hMSCs have been shown previously against *P. aeruginosa* and *Escherichia coli* and linked to the secretion of antimicrobial peptides.^51^ It was anticipated, therefore, that Ti surfaces with a better spread of hMSCs and a greater proportion of viable cells would better protect against *P. aeruginosa* biofilm formation, and indeed, this was observed. Fluorescence microscopy revealed, a confluent *P. aeruginosa* biofilm formed within 24 h of co-culture on uFlat surfaces, whereas far fewer bacteria were observed on uNN, although hMSCs were poorly spread on both surfaces. However, on the coated surfaces, the hMSCs were better spread while bacterial biofilm formation was suppressed. Together, these data indicated that the PEA+FN+BMP2 coating simultaneously promoted hMSC adhesion, counteracting the antiadhesive effects of the nanotopography, and preserved the antibacterial properties of the nanotopographies (Figures 2c and 3a). Moreover, these data suggested that the presence of established, well-spread, hMSCs in combination with the coated nanotopographies exhibited synergistic antibacterial action to prevent biofilm formation and reduce bacterial adhesion levels.

To investigate this cooperative behavior in molecular detail, a metabolomics approach was used to identify the up- and down-regulation of secreted metabolites (from both bacterial and hMSC metabolites), hMSC intracellular metabolites, and bacterial intracellular metabolites in response to the co-cultures on the coated nanotopography. For *P. aeruginosa*, QSMs 3-oxo-C10-HSL, 3-oxo-C12-HSL and 3-oxo-C14-HSL (from the LasR system) were again identified by high-resolution mass spectrometry, but not QSMs from the RhlR or quinoline systems. Compared to the uFlat control, QSMs, including those that regulate the LasR system, were seen to be elevated in sessile *P. aeruginosa* on cFlat and uNN in co-culture (Figure 4c). Importantly, a general trend of QSM down-regulation was observed for *P. aeruginosa* co-cultured with hMSCs on cNN, including for 3-oxo-C12-HSL and 3-oxo-C14-HSL (Figure 4c). This was notable, as inhibition of LasR induction is a biofilm prevention strategy.^40,52−54^

QSMs produced by planktonic bacteria in the surrounding media were also evaluated. Only two QSMs were detected (2-oxo-C10-HSL and C5-HSL-D9), which, for the cFlat, uNN and cNN surfaces, were found at lower levels than determined for *P. aeruginosa* co-cultured on the uFlat surface (Figure S22). None of the other QSMs in the library were detected (Table S4), indicating that hMSCs cultured on the u/cFlat and u/cNN surfaces were able to restrict the activity of this other bacterial population despite not being in direct contact.

To study the effects of co-culture on the hMSCs, monocultured and co-cultured hMSCs were compared using metabolomics through liquid chromatography mass spectrometry. Metabolites were extracted from the media (secretome) using a 1:3:1 chloroform:methanol:water buffer, accepting that, for the co-culture, these samples would be a combination of metabolites from both hMSCs and *P. aeruginosa*. Intracellular metabolites from hMSCs were also extracted (with no bead beating so as not to include bacterial metabolites from the co-culture). Our hypothesis was that different metabolites seen for hMSCs in co-culture versus monoculture on the antibacterial, coated nanotopographies would be involved in combating bacterial infection.^55^ Furthermore, we hypothesized that such metabolites could have potential to further reduce bacterial biofilm formation when exogenously supplemented into co-cultures.

We classically think of metabolites for use as biomarkers, e.g., glucose and lactate measurements.^56,57^ However, metabolites can also drive desirable processes, including targeted growth and differentiation of hMSCs.^55,58−61^ Therefore, here, for the first time, we wanted to determine whether these metabolites could control the activity of different populations of cells.

The 12 intracellular metabolites with the greatest changes in abundance in the hMSCs monoculture and co-culture are shown in Figure 4d. Further descriptions of the metabolite family and pathways involved are presented in Table S5. Two of the metabolites identified as elevated in co-culture compared to monoculture were glutathione (GSH) and citrate. GSH is a potent antioxidant that helps prevent damage on cells (cellular senescence and apoptosis) from reactive oxygen species (ROS).^62^ In this case, GSH was elevated in hMSCs in the presence of bacteria on both flat and NN surfaces, with or without coating, implying that the production of GSH by the hMSCs was triggered by *P. aeruginosa*. Indeed, it has been hypothesized that bacteria can induce oxidative stress in infections.^63^ A similar pattern was seen for citrate and therefore thought to be involved in the cell response to the presence of bacteria. Citrate takes part in carbohydrate metabolism^64^ and is a metabolite in the citric acid cycle (or Krebs cycle).^64^ Interestingly, sodium citrate has previously also been found to be detrimental to *P. aeruginosa* biofilm establishment.^65^ Moreover, for hMSC secreted metabolites in co-culture, the molecule with the greatest change was 2-oxoglutamarate, which is a biproduct of the citrate cycle (Figure 4e). The other modulated metabolites were related to cell amino acid metabolism (Table S6).

Given that citrate and 2-oxoglutamarate production was changed by hMSCs in co-culture, we next explored the potential for citrate to be exploited as an antibacterial agent.^65^ We selected citrate as it was found in greater abundance as an intracellular metabolite and, therefore, could be hypothesized to be a metabolite the cells were using to achieve differentiation in the presence of bacteria, likely through increased citric acid cycle use to generate energy.^64,66^ 2-Oxoglutamarate is a byproduct of citric acid cycle and GSH is likely increased in response to citric acid cycle activity due to increased ROS generated during mitochondrial respiration.^67^ Thus, citrate appears canonical to the process and likely drives desirable activity as an intracellular metabolite. Therefore, considering this and its potential antibiofilm activity, we decided to test citrate as an activity metabolite and feed it to the cultures.

Initially, the viability of hMSCs to withstand citrate concentrations from 0.25% to 4% (w/v) was examined (Figure S23). Concentrations that were nontoxic to hMSCs, 0.25 and 0.5% (w/v), were then tested on *P. aeruginosa* at 10^3^ CFU overnight, with and without the use of 0.3% antibiotic P/S. After 24 h incubation, the population of *P. aeruginosa* incubated with the combination of 0.25 and 0.5% concentrations of citrate and P/S was significantly reduced to about 50% and 45% respectively, while the citrate on its own reduced the bacteria population to about 60–70% for both tested concentrations (Figure 4f). Furthermore, no significance was found when comparing the usual 0.3% P/S with 0.5% citrate, indicating that citrate could directly mediate antibacterial effects against *P. aeruginosa*.

The ability of hMSCs to differentiate was further assessed in the presence of citrate. After 14 days, there was an increased trend in the mRNA expression of Runx2 (Figure S24), which shows that osteogenesis is at least not detrimentally affected by citrate and is potentially supported. We note that citrate is present in the bone *in vivo* microenvironment, and it has been proven that supplementation of this metabolite *in vitro* can increase osteogenesis in MSCs.^66^

## Conclusion

In this study, we have demonstrated that active protein-based coatings on Ti nanotopographies can improve hMSC adhesion and combat biofilm–forming bacteria. This was achieved by exploiting the ability of PEA to unfold FN and expose its integrin binding sites to enhance cell adhesion and to present BMP2 in solid phase to provide synergistic integrin-growth factor signaling.^21,22^

The coating on the Ti nanotopography surfaces is reproducible, did not hinder the nanotopography structures, and enhanced the hydrophilicity of the surfaces. Furthermore, the coating enhanced hMSC adhesion (and adhesion-related signaling) and supported cell viability, proliferation, and differentiation.

Importantly, the Ti nanotopographies with PEA+FN+BMP2 coating reduced *P. aeruginosa* biofilm formation and induced the downregulation of virulence factors such as cell surface appendages and QSMs, particularly cNN. Furthermore, the changes in the hMSC metabolome and secretome in co-culture with *P. aeruginosa* allowed us to identify active metabolites, notably citrate, that reduce biofilm formation while helping hMSC differentiation.

Our results support a platform that can be used to understand hMSC-biofilm dynamics and effects on QS. This model can also be utilized to identify novel bioactive metabolites that can act as antibacterial adjuncts. Further, the use of Ti nanotopographies with pro-osteogenic, antibiofilm properties could help develop novel orthopedic implant materials where infection is a risk.

## Materials and Methods

All materials were acquired from Sigma, unless otherwise stated.

### Titanium Nanotopography Synthesis

Titanium (Ti) discs (⌀ = 11 mm, grade 1), were polished to grit levels of 4000 using Struers tegraPol-15. The discs were then cleaned by sonication (Grant XUB5) for 15 min in dH_2_O and immersed in absolute ethanol (Merk) for 10 min before blow-dried with compressed air. The discs were placed in upright position using custom-made PTFE holder and immersed in a beaker containing prewarmed 2 M sodium hydroxide (NaOH) (Fisher) solution at 60 °C. The nanospikes surface (NS) was generated by etching the Ti discs for 2 h while the nanonetwork surface (NN) was etched for 16 h. Then, the discs were washed thoroughly by using dH_2_O and 100% ethanol (Merck) before being left to dry overnight. The final step involved placing the discs in the chamber furnace for calcination for 2 h at 600 °C with a heating rate of 10 °C per min. The discs were cooled and stored in a sterile, enclosed plastic Petri dish until use.

### Plasma Polymerized Ethyl Acrylate Coatings

The coatings of Ti surfaces were carried out following the optimized procedures from Damiati et al.^8^ Briefly, a layer of plasma polymerized ethyl acrylate (PEA)^68^ was deposited on the surfaces for 90 s at 100 W. Human fibronectin (FN) (F2006–2MG) was added on the Ti surfaces by adding 200 μL of 20 μg·mL^–1^ FN/PBS solution for 1 h, followed by 200 μL of 1% BSA/PBS for 30 min to block nonspecific sites. The samples were washed with PBS, and 200 μL of BMP2 (14791–10UG) was added for 1 h at 100 μg·mL^–1^.

### Atomic Force Microscopy

Atomic Force Microscopy (AFM) was used to measure and observe the roughness of the Ti nanostructures. These were performed using AFM multimode operated in tapping mode in air, equipped with NanoScope IIIa controller from Veeco (Manchester, UK) using NanoScope 5.30r2 software. An area of 5 μm × 5 μm with a scan rate of 0.5 Hz. Three scans were performed per sample and the height were quantified using JPK Nanowizard software.

### Plasma Polymerized Ethyl Acrylate Coating Thickness

Coverslips were submitted to plasma polyethyl acrylate (PEA) coating for 90 s at 100 W. A sharp blade was used to create a scratch on the PEA coated coverslips. AFM was used to measure the scratch height, a line was traced over the measured height, and the transverse section was measured as shown.

### X-ray Photoelectron Spectroscopy

The chemical composition of the nanostructures before and after PEA coating was analyzed by using X-ray photoelectron spectroscopy (XPS). This was done in collaboration with Harwell XPS. A K-alpha apparatus (ThermoFisher Scientific) was used with a microfocused monochromatic Al Kα source (X-ray energy of 1486.6 eV) using 12 kV, 3 mA, 36 W of power, and 400 × 800 μm spot size. CasaXPS 2.3.16 software was used for the spectral analyses.

### Wet Contact Angle

Sessile drop contact angle was correlated to the wet contact angle (WCA) of the Ti surfaces. Milli Q water droplets (3 μL) were added to the nanotopographies in different locations of the Ti disc, and the contact angle was measured (Optical Tensiometer Theta, Biolin Scientific).

### Protein Surface Coating Quantification

The quantification of adsorbed protein was performed by calculating the amount of protein left in the supernatant after coating and then subtracting this from the original stock concentration. FN and BMP2 levels in the supernatants were calculated using enzyme-linked immunosorbent assay (ELISA) duo-set Human Fibronectin (DY1918, R&D systems), and Human BMP2 (DY355, R&D systems), respectively, following instructions from the manufacturer.

### Bacterial Assays

*P. aeruginosa* was cultivated overnight in DMEM at 37 °C, 200 rpm. Suspensions were adjusted to OD_600_ 0.1 and then grown to OD_600_ 0.3 (equivalent to 10^8^ CFU·mL^–1^), before being diluted to 10^3^ CFU·mL^–1^ for all experiments in co-culture media (DMEM supplemented with 1% fetal bovine serum (FBS), 2.2 U·mL^–1^ penicillin/streptomycin [0.3% of total solution], 1% Eagle’s minimum essential medium nonessential amino acid solution (MEM NEAA, Gibco), 1% l-glutamine, and 1% sodium pyruvate).

### Scanning Electron Microscopy

Samples were fixed using 2.5% glutaraldehyde in 0.1 m sodium cacodylate and rinsed twice with sodium cacodylate buffer. 1% osmium tetroxide was used for membrane contrast for 1 h and washed using distilled water three times for 10 min. A series of dehydration with ethanol were done with 30%, 50%, 70%, 95%, and 100%, and hexamethyldisilane was used for complete drying of the samples. The Ti discs or coverslips were mounted in stubs using carbon tape and sputter coated using gold/palladium at 20 nm thickness. Samples were imaged using a JEOL IT100 SEM, and Carl ZEISS SEM.

### Bacterial Viability Live/Dead

*P. aeruginosa* was cultured as in the previous section. One mL of the bacteria suspension at 10^3^ CFU in co-culture media was added to the Ti Flat, NS, and NN coated or uncoated, and incubated in a humidified incubator at 37 °C under a 5% carbon dioxide atmosphere. The next day, the samples were stained using BacLight 1:1000 for each component and imaged in an inverted fluorescent microscope (EVOS S7000, ThermoFisher, UK). The obtained images were analyzed using Fiji software (NIH, USA). Images were converted to 8 bit and a threshold was applied before the living cells (green) were quantified using the Analyze Particles function.

### Metabolite Extraction for *P. aeruginosa*

Metabolites were extracted using a cold extraction buffer containing 1:3:1 chloroform, ethanol, and water. Planktonic bacteria and biofilm bacteria were extracted separately. The Ti discs were carefully removed from the well plate to avoid disrupting any bacterial cells on the biofilm and placed in 5 mL bijoux containing 1 mL of PBS before being sonicated for 10 min to detach bacteria from the surfaces. The remaining 1 mL of planktonic bacteria were aspirated from the wells and added to an Eppendorf tube. All suspensions were then centrifuged at 7000 *g* for 5 min to pellet the bacteria. The pellet was resuspended in extraction buffer and transferred to an Eppendorf tube containing 1 g of sterilized acid-washed beads (68772). The bacteria were lysed using a bead beater (Fisherbrand Bead Mill 24) using three 30 s cycles each with 30 min in between each cycle. The tubes were then centrifuged, and the supernatant was sent for analysis to EdinOmics at the University of Edinburgh.

### Metabolite Quantification of *P. aeruginosa* in Monoculture

Briefly, the HPLC column was a 2 mm × 10 cm Waters BEH C18 column with a flow rate of 250 μL per minute. The mobile phase A was water plus 0.1% formic acid. B was acetonitrile with 0.1% formic acid. Gradient held at 10% B for 1 min, then ran from 10% B to 50% B in 0.5 min, then to 99% B in 4 min, held at 99% B for 4.5 min, and equilibrated at 10% B for 5 min, for a total run time of 15 min. Mass spectrometry was performed with a Quantiva Triple Quadrupole mass spectrometer (Thermo, Hemel Hempstead). Voltage used for both positive and negative ionization was 3.5 kV. Sheath gas was maintained at 35 and aux gas was set to 1. Ion transfer tube and vaporizer temperatures were set to 325 and 275 °C, respectively.

### Seeding hMSCs onto the Surfaces

Ethics for mesenchymal stromal cell extraction were granted to our collaborators at the University of Southampton: Prof. Richard Oreffo, NRES number: 194/99/1, LREC number: 31875.

hMSCs were cultured in a T175 flast (Corning) using DMEM supplemented with 10% fetal bovine serum (FBS), 1% penicillin/streptomycin, 1% Eagle’s minimum essential medium nonessential amino acid solution (MEM NEAA, Gibco), 1% l-glutamine, and 1% sodium pyruvate), until 80% conlfuent. The cells were then detached from flasks using 0.5% trypsin/versene solution and counted.

Ti discs coated with PEA, FN, and BMP2 were placed in a 24-well plate. 10,000 cells were added to each Ti disc (Flat, NS, NN) or coverslip with and without coatings in seeding media (DMEM containing all supplements, and 2% FBS, 0.3% penicillin/streptomycin) overnight. The media was changed to 1% co-culture media (DMEM containing all supplements, and 1% FBS, 0.3% penicillin/streptomycin) the next day. The low percentage of antibiotic was chosen for all experiments so that hMSCs and bacteria were in the same type of media.

### Immunofluorescence Staining

Samples were fixed using 4% paraformaldehyde/PBS for 15 min at 37 °C, and permeabilized using 0.5% Triton X-100 buffer containing 5 mmol sodium chloride, 63 μmol magnesium chloride, 2 mmol HEPES, pH 7.2 in PBS, for 5 min at 4 °C. Nonspecific binding sites were blocked for 5 min using a 1% bovine serum albumin (BSA) solution in PBS at room temperature. Immunolabeling with primary antibody (1:100) and phalloidin rhodamine (1:500) was overnight at 4 °C. The samples were washed using 0.5% Tween-20 solution in PBS three times for 5 min at room temperature with shaking. The secondary biotinylated antibody was added (1:100) and incubated for 1 h at 37 °C. The samples were washed as before, and (1:100) streptavidin fluorescein antibody was added for 30 min at 4 °C. Finally, samples were washed and mounted onto a glass coverslip using Vectashield containing DAPI (Vector laboratories) and imaged using an EVOS inverted fluorescence microscope (ThermoFisher, UK).

### hMSC Viability

24h after hMSCs were seeded on the nanotopography surfaces, a live/dead assay (L3224, ThermoFisher, UK) was used to measure viability. The cell monolayer on the surfaces was washed twice with warm PBS. 2 μm calcein AM and 4 μm ethidium homodimer-1 were dissolved in fully supplemented DMEM and 500 μL was added per sample before incubation for 15 min in the dark at room temperature. The samples were then transferred to a well plate and imaged in PBS by using an inverted fluorescent microscope (EVOS 7000, ThermoFisher, UK). The obtained images were analyzed using Fiji software (NIH, USA). Images were converted to 8 bit and a threshold was applied before the living cells (green) were quantified using the Analyze Particles function.

### In-Cell Western

Samples were fixed using 4% paraformaldehyde/PBS for 15 min at 37 °C, and permeabilized using 0.5% Triton X-100 buffer containing 5 mM sodium chloride, 63 μM magnesium chloride, 2 mM HEPES, pH 7.2 in PBS, for 5 min at 4 °C. Blocking was done for 1.5 h using 1% milk solution in PBS at room temperature in shaking motion. Primary antibody was added (1:100) overnight to a 1% milk solution. Samples were washed using 0.1% Tween-20 in PBS 5 times for 5 min in shaking motion. Secondary antibody and CellTag were added (1:1000, and 1:500 respectively) for 1 h at room temperature. After incubation, samples were washed as before and air-dried before reading. The samples were imaged using a Licor Odyssey M.

### Differentiation on Titanium Nanotopographies

hMSCs on Ti nanosurfaces were cultured for 14 days in co-culture media (containing 1% FBS and 0.3% penicillin/streptomycin). The content of the two discs was pooled to have sufficient RNA. The RNA was isolated using a RNeasy kit (74104, Qiagen) following the instructions of the manufacturer.

### Quantitative PCR

Total RNA was extracted and purified using an RNeasy Micro Kit (Qiagen). The purified RNA was immediately processed for cDNA synthesis using a QuantiTect Reverse Transcription Kit (Qiagen). *GAPDH* and *RPL13A* were used as housekeeping genes. SYBR Green dye was used to target the synthesized cDNA. Real-time PCR was performed using 10 ng of cDNA per well. The samples were compared to Runx2 and the osteopontin genes (see Table 1.

**Table 1:** 

Gene	Forward	Reverse
RUNX2	CCCAGTATGAGAGTAGGTGTCC	GGGTAAGACTGGTCATAGGACC
Osteopontin	AGCTGGATGACCAGAGTGCT	TGAAATTCATGGCTGTGGAA
GAPDH	TCAAGGCTGAGAACGGGAA	TGGGTGGCAGTGATGGCA
RPL13A	CTCAAGGTGTTTGACGGCATCC	TACTTCCAGCCAACCTCGTGAG

### hMSC and *P. aeruginosa* Co-Culture

hMSCs were detached from flasks using 0.5% trypsin/versene solution and counted.

Ti discs coated with PEA, FN, and BMP2 were placed in a 24-well plate. hMSC cells (10,000) were added to each Ti disc (Flat, NS, NN) or coverslip in seeding media (DMEM containing all supplements and 2% FBS, 0.3% penicillin/streptomycin) overnight. The next day, the medium was removed and the cell monolayer was washed with PBS once before adding 1 mL of *P. aeruginosa* in co-culture media.

### Metabolite Quantification for *P. aeruginosa* in Co-Culture with hMSCs

The instrumentation consisted of an Agilent 1290 Infinity II series ultrahigh performance liquid chromatography system coupled to an Agilent 6560 ion mobility quadrupole time-of-flight mass spectrometer with a Dual Agilent Jet Stream (AJS) electron ionization source (ESI). Chromatographic separation was performed using a ZORBAX Extend-C18 rapid resolution HT 2.1 mm × 50 mm, 1.8 μm column (Agilent Technologies 727700–902, Santa Clara CA). The solvent system consisted of MS-grade water with 0.1% formic acid as solvent A and MS-grade acetonitrile with 0.1% formic acid as solvent B. The solvent gradient was set to a constant flow rate of 0.150 mL·min^–1^ starting at 90% of solvent A, which was maintained for 1 min. The gradient was dropped to 50% solvent A at 1.5 min and to 1% solvent A at 5.5 min, and it was maintained until 7 min. The gradient was increased back to starting conditions of 90% solvent A at 7.9 min, where it remained until 9.9 min. The column was maintained at a constant temperature of 40 °C throughout the run. Five μL portion of each sample was injected into the column for analysis, and a quality control sample (generated by pooling equal volumes of each extract) was injected after every five samples to monitor instrument performance throughout data acquisition. Data were acquired in positive ionization mode by scanning a mass range of 50–1500 *m*/*z* with an acquisition rate of 1 spectra s^–1^. The Dual AJS ESI gas temperature was maintained at 325 °C at a flow rate of 13 L min^–1^. The nozzle voltage was set to 2000 V and VCap to 3750 V. Data acquisition and processing were performed using the Agilent MassHunter software suite. Standards were run alongside the samples, and [M + H]+ ion species were used to identify the HSLs, using accurate mass, drift time, collision cross section, and chromatographic retention time parameters.

### Metabolite Extraction for hMSCs in Mono- and Co-Culture with *P. aeruginosa*

The metabolite extraction from hMSCs was performed using extraction buffer containing 1:3:1 chloroform, methanol, and water. 25 μL of culturing media were added to 1 mL of extraction buffer. The cell monolayers on the Ti surfaces were placed in a 24 well plate containing 1 mL of extraction buffer. All samples were placed in a shaking stage for 1 h at 4 °C to release and quench all metabolites. The extraction buffer containing metabolites was placed in a fresh Eppendorf tube and centrifuged at 7000 *g* for 15 min at 4 °C. The supernatant was put into a fresh Eppendorf caring not to take any pelleted debris and sent for analysis to the Polyomics facility at the University of Glasgow.

Cleared extracts were used for hydrophilic interaction liquid chromatography–mass spectrometry analysis using Orbitrap Exactive with UltiMate 3000 RSLC (Rapid separation liquid chromatography, ThermoFisher), and a 150 mm × 4.6 mm ZIC-pHILIC for hydrophilic interaction LC with a flow of 300 μL per minute. Sample protein concentrations were measured by Nanodrop and used to standardize samples where required. A standardized pipeline, consisting of XCMS (peak picking), MzMatch (filtering and grouping), and IDEOM file with raw data generated for postprocessing filtering and identification. Target metabolites identified were validated against a panel of unambiguous standards by mass and predicted retention time. Further putative identifications were generated by the mass and predicted retention times.

## Data Availability

Data available from DOI 10.5525/gla.researchdata.1610 through the University of Glasgow.

## Supporting Information Available

Additional experimental details, materials and methods, including atomic force microscopy images and measurements, X-ray spectroscopy from nanotopographies before and after coating, wet contact angle measurements, antibiotic titration, adenosine triphosphate analysis for bacterial culture, cell area and perimeter of hMSCs, fluorescence microscopy of hMSC focal adhesions, electron microscopy images from co-cultures, live/dead images of hMSCs in co-culture with *P. aeruginosa,* cell area and perimeter of hMSCs in co-culture, identified QSMs in planktonic *P. aeruginosa* after co-culture with hMSCs, the effect of citrate on hMSCs, the effect of citrate on hMSC differentiation. Supplementary tables including list of QSMs, list of QSMs screened in *P. aeruginosa* after incubation on nanotopographies, the top 12 changes in the metabolome map family for hMSCs. Additional methods for the Supporting Information data (PDF)
