# Supplementary material for: Nanotopography Influences Host–Pathogen Quorum Sensing and Facilitates Selection of Bioactive Metabolites in Mesenchymal Stromal Cells and Pseudomonas aeruginosa Co-Cultures
Source: ACS Appl Mater Interfaces. 2024 Aug 8;16(33):43374–86. doi: 10.1021/acsami.4c09291 (PMC11345723; doi:10.1021/acsami.4c09291)
Supplement: Supplementary file 1 — am4c09291_si_001.pdf [file am4c09291_si_001.pdf]

Supporting Information

Nanotopography influences host-pathogen quorum sensing and facilitates selection of bioactive metabolites in mesenchymal stromal cells and *Pseudomonas aeruginosa* co-cultures

Rosalia Cuahtecontzi Delint[1*], Mohd I. Ishak[2], Penelope M. Tsimbouri[1], Vineetha Jayawarna[1], Karl V. Burgess[3], Gordon Ramage[4], Angela H. Nobbs[2], Laila Damiati[5], Manuel Salmeron-Sanchez[1], Bo Su[2], & Matthew J. Dalby[1].

[1]Centre for the Cellular Microenvironment, School of Molecular Biosciences, College of Medical, Veterinary and Life Sciences, Mazumdar-Shaw Advanced Research Centre, University of Glasgow, Glasgow, G11 6EW, UK

[2]Bristol Dental School Research Laboratories, Dorothy Hodgkin Building, University of Bristol, Bristol BS1 3NY, UK.

[3]EdinOmics, University of Edinburgh, Max Born Crescent, Edinburgh, EH9 3BF, UK.

[4]Safeguarding Health through Infection Prevention (SHIP) Research Group, Research Centre for Health, Glasgow Caledonian University, Glasgow, G4 0BA, UK.

[5]Department of Biological Sciences, College of Science, University of Jeddah, Jeddah 23218, Saudi Arabia.

***Corresponding author email:**
Rosalia.cuahtecontzidelint@glasgow.ac.uk

Keywords
Nanotopography, active coatings, antibacterial, quorum sensing molecules, metabolomics.

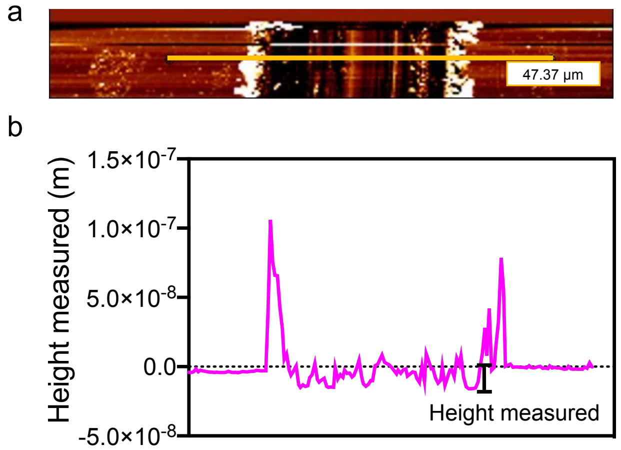

c

Average Height Measured
19.44 nm ± 1.2

Figure S1. Representative atomic force microscopy (AFM) images to measure coating thickness. Coverslips were submitted to plasma polyethylacrylate (PEA) coating for 90 seconds at 100 Watts and a sharp blade used to create a scratch on the PEA coated coverslips. **a** AFM was used to measure the scratch height. **b** A line was traced over the measured height and the transverse section was measured as shown. **c** The average of three different coverslips was measured and averaged.

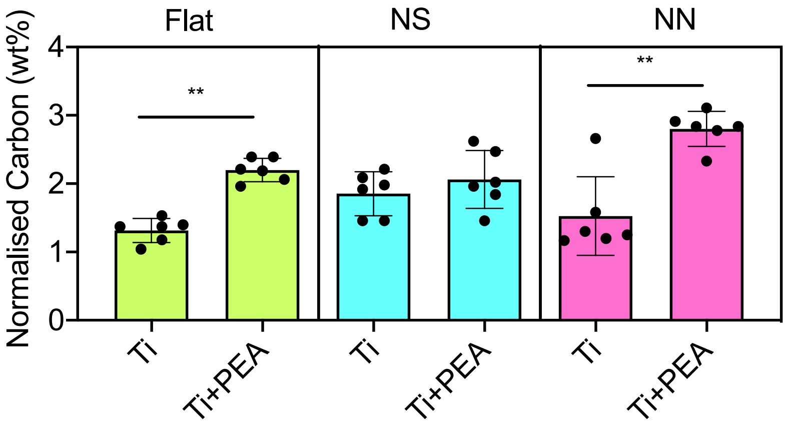

Figure S2. **Energy dispersive X-ray spectroscopy from flat, NS, and NN surfaces before and after being coated with PEA.** A Zeiss Gemini SEM microscope was used to detect the carbon from the topographies before and after PEA coating on flat (green), NS (blue), and NN (pink) surfaces. Six different points were assessed on each disc. Data shown as normalised weight percentage of carbon. Comparison of differences was tested using Mann-Whitney test with a p-value <0.001 (**) considered highly significant.

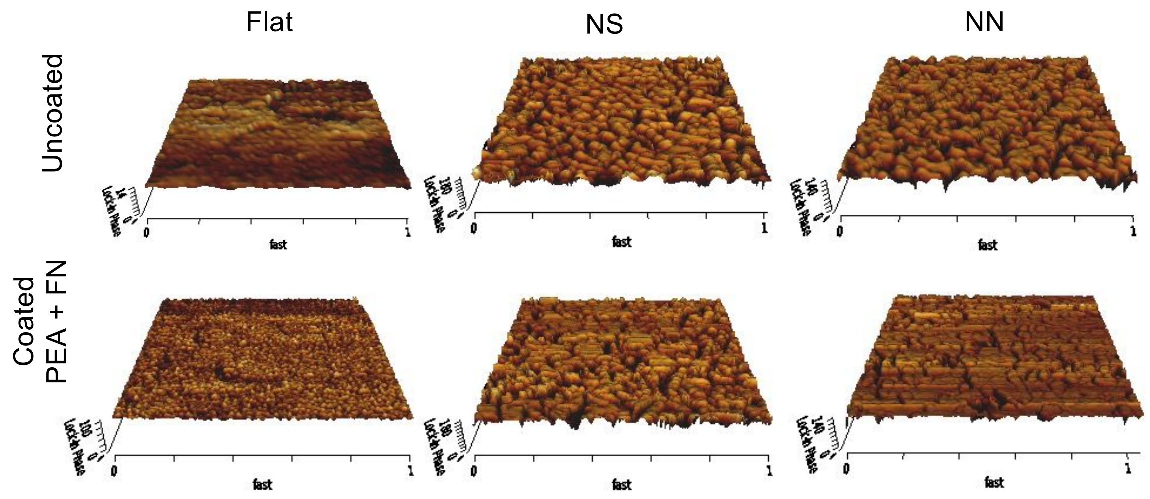

Figure S3. AFM images from nanotopographies. 1x1 micrograph images from AFM on Flat, NS, and NN surfaces before and after being coated with PEA and FN.

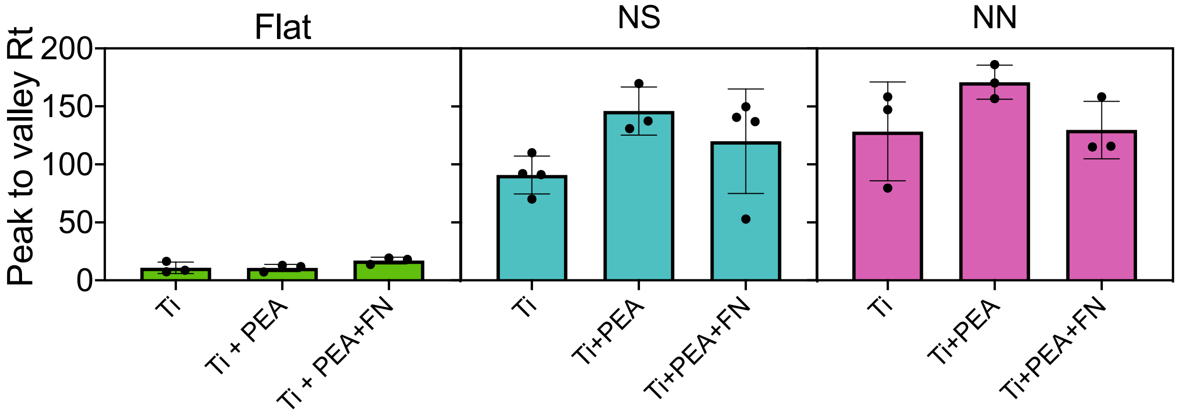

Figure S4. Peak to valley (Rt) measurements from titanium surfaces. Rt values from uncoated Flat (green), NS (blue), and NN (pink) Ti surfaces, after coating using PEA, and after fibronectin addition and drying flat (green), NS (blue), and NN (pink). Average represented as bars with individual values and standard deviation. Comparison of differences was tested using a Kruskal-Wallis test.

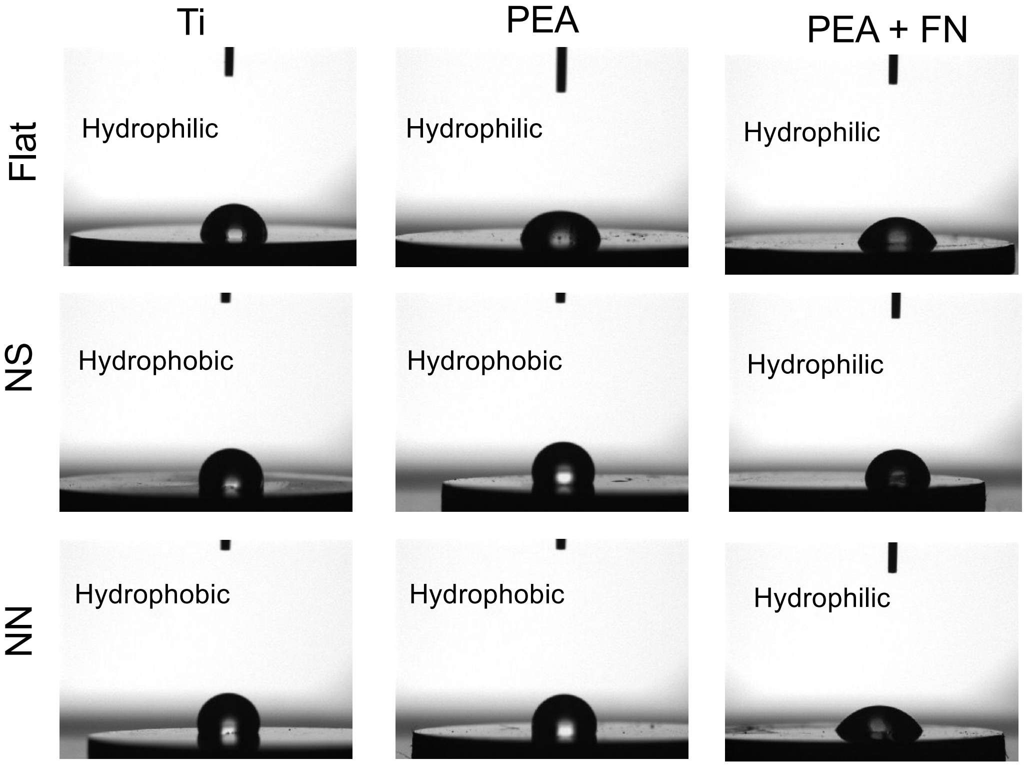

Figure S5. Representative wet contact angle measurement (WCA) images. Titanium Flat, NS, and NN samples were submitted to plasma polyethylacrylate (PEA) coating for 90 minutes at 100 W. Sessile drop contact angle was correlated to the WCA. A 3 μL droplet of Milli Q water was added, and the contact angle measured. An angle higher than 90 degrees was considered hydrophobic.

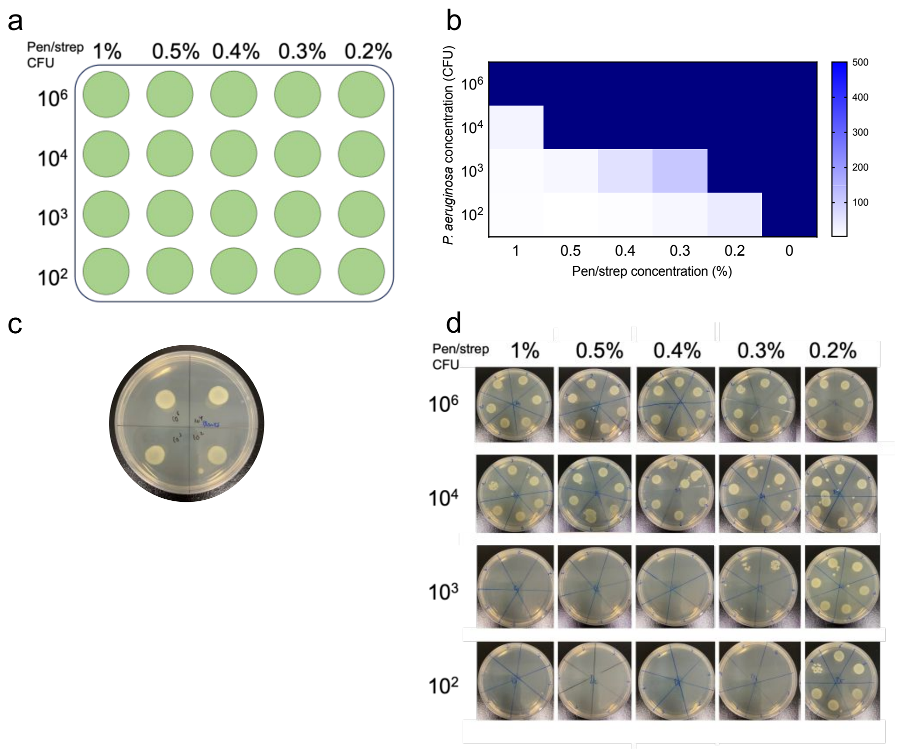

Figure S6. Antibiotic and CFU titration for co-culture experiments. **a** *P. aeruginosa* was cultured in different CFU gradients (from 10^8 to 10^2) and penicillin/streptomycin (from 1% to 0.2%) for 24 hours in DMEM. **b** The next day, bacterial viability was assessed using BacTiter-Glo. Mean values are represented as a heatmap using a blue colorimetric scale, with darker blue meaning a higher intensity reading and therefore greater viability. **c** From the same cell suspensions, untreated controls were plated, and **d** bacterial suspensions were serially diluted from 10^{-2} to 10^{-7} to assess cell survival after a further 24 hours .

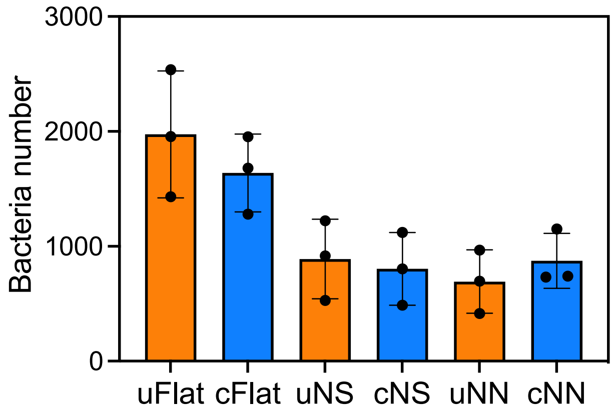

Figure S7. Quantification of total bacteria attached to nanotopographies. *P. aeruginosa* was cultured overnight on the nanotopographies using co-culture media and stained using Live/Dead stain. After microscopy assessment, the total number of attached bacteria were quantified using Fiji software. Statistics were performed using a Kruskal-Wallis test.

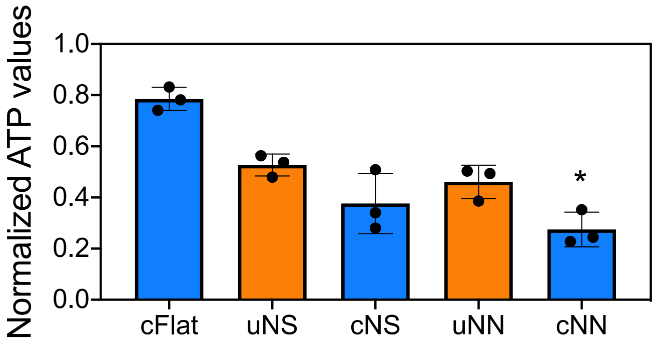

Figure S8. ATP quantification following bacterial culture on titanium nanotopographies. *P. aeruginosa* was seeded at 10^3 CFU under a 5% CO_2 atmosphere at 37 °C on Ti Flat, NS, NN surfaces, coated or uncoated. After 24 hours, levels of ATP were quantified by BacTiter Glo assay. Comparison of differences was tested using Kruskal-Wallis test.

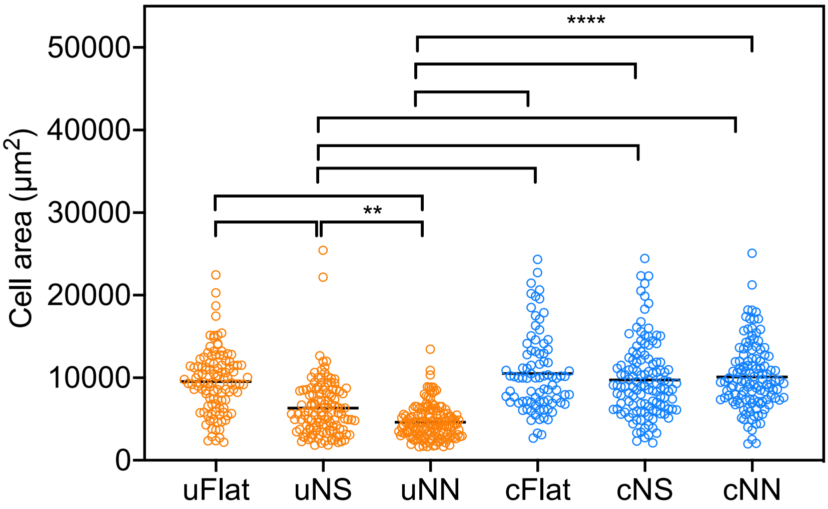

Figure S9. Cell area quantification from human mesenchymal stromal cells (hMSC) on titanium nanotopographies. hMSCs were seeded overnight on Flat, NS and NN titanium surfaces with and without coatings. A live/dead assay was performed and the living hMSCs were quantified and analyzed for area using Fiji software. Comparison of differences was tested using Kruskal-Wallis test with a p-value <0.005 (**) considered significant and p-value <0.0001 (****) considered highly significant. All data with brackets has a significance of **** except where ** was placed.

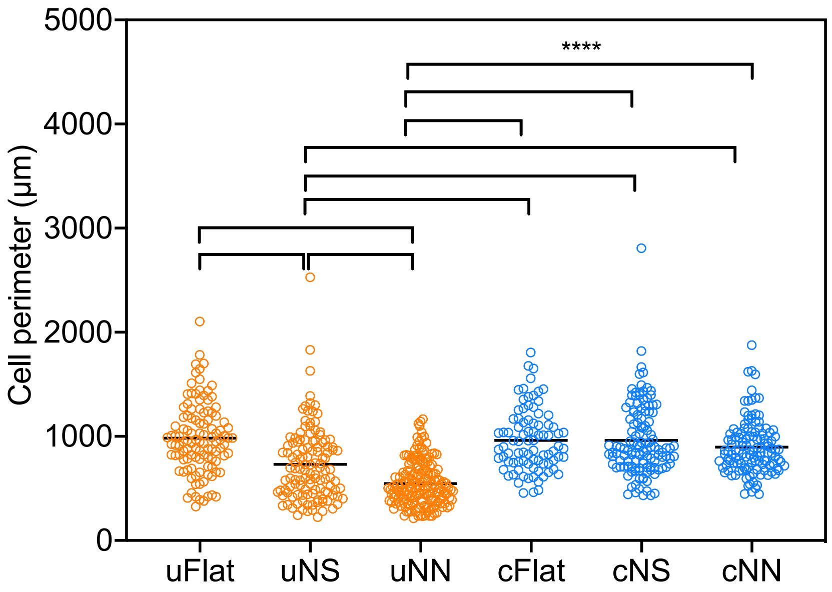

Figure S10. Cell perimeter quantification from human mesenchymal stromal cells (hMSC) on titanium nanotopographies. hMSCs were seeded overnight on Flat, NS and NN titanium surfaces with and without coatings. A live/dead assay was performed and the living hMSCs were quantified and analyzed for perimeter using Fiji software. Comparison of differences was tested using Kruskal-Wallis test with a p-value <0.0001 (****) considered highly significant. All data with brackets has a significance of ****.

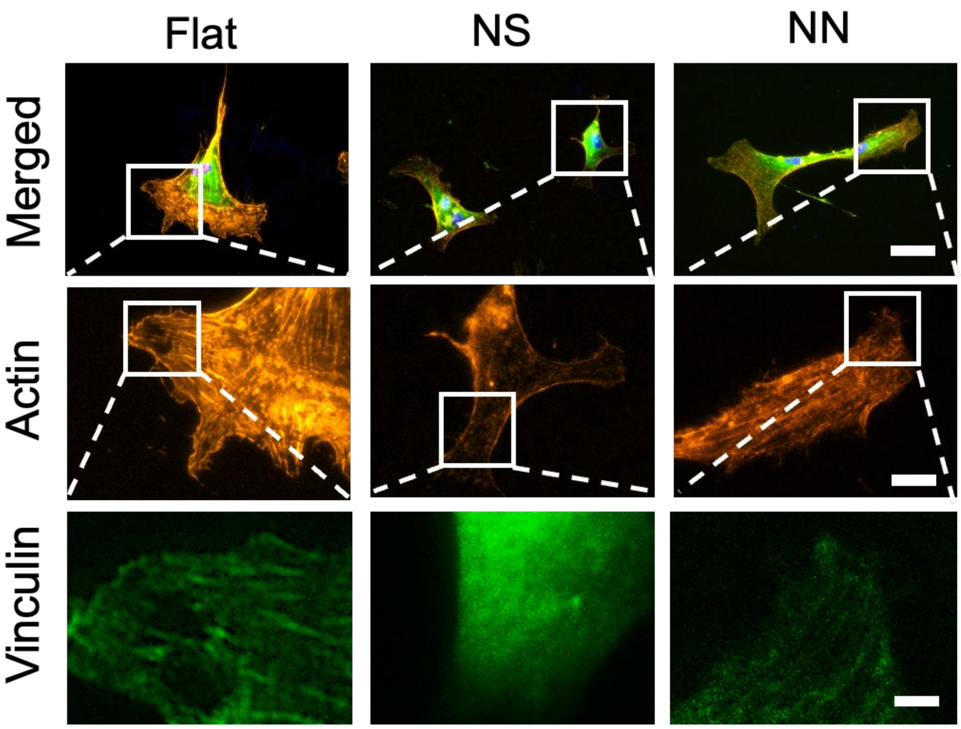

Figure S11. Representative images from fluorescence microscopy of human mesenchymal stromal cells (hMSCs) for focal adhesions. hMSCs were incubated overnight on Flat and NN titanium surfaces without coatings. The next day, the samples were fixed, and stained for actin (orange) and vinculin (green). Samples were observed using an inverted fluorescence microscope EVOS ThermoFisher. Scale bars from top, middle, bottom panels: 100, 30, and 10 μm.

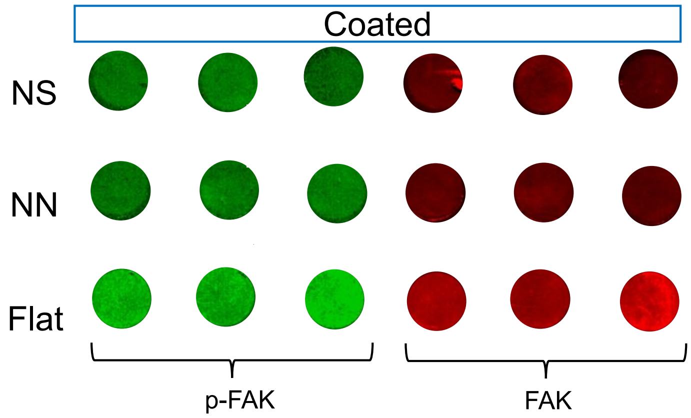

Figure S12. Representative images from in-cell western assay for p-FAK ratio with FAK. hMSCs were incubated on flat, NS, or NN nanotopographies coated or uncoated, using co-culture media for 24 hours under a 5% CO_2 atmosphere at 37°C. Samples were fixed, stained for protein expression, read using a Licor Odyssey M, and analyzed using Empiria software.

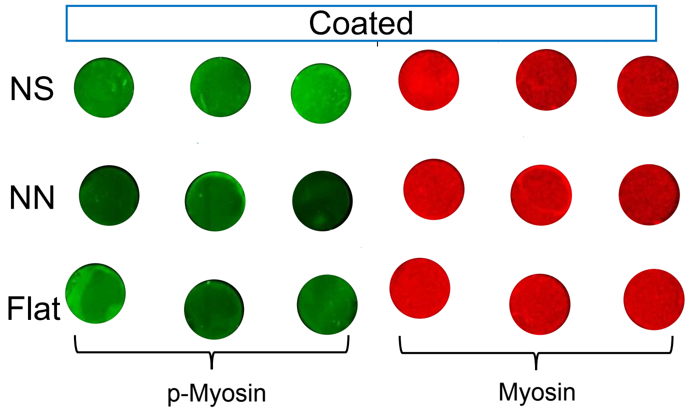

Figure S13. Representative images from in-cell western assay for p-myosin ratio with myosin. hMSCs were incubated on flat, NS, or NN nanotopographies coated or uncoated, using co-culture media for 24 hours under a 5% CO_2 atmosphere at 37°C. Samples were fixed, stained for protein expression, read using a Licor Odyssey M, and analyzed using Empiria software.

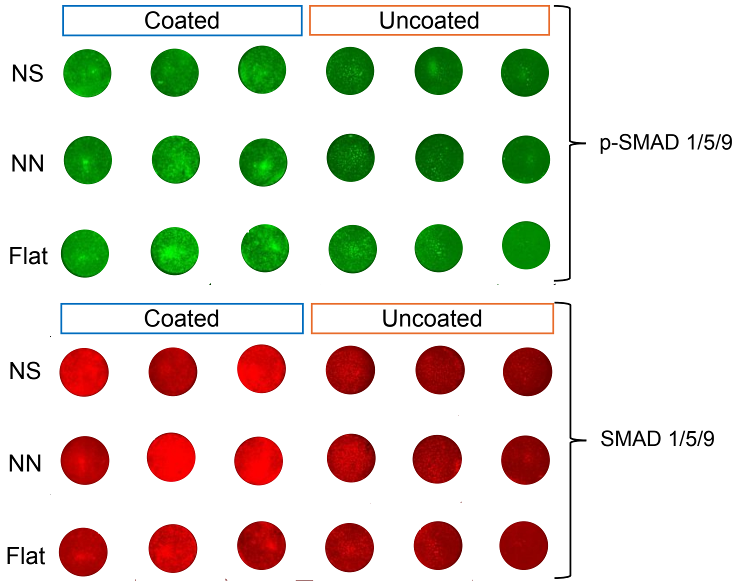

Figure S14. Representative images from in-cell western assay for p-SMAD 1/5/9 ratio with SMAD 1/5/9. hMSCs were incubated on flat, NS, or NN nanotopographies coated or uncoated, using co-culture media for 24 hours under a 5% CO_2 atmosphere at 37°C. Samples were fixed, stained for protein expression, read using a Licor Odyssey M, and analyzed using Empiria software.

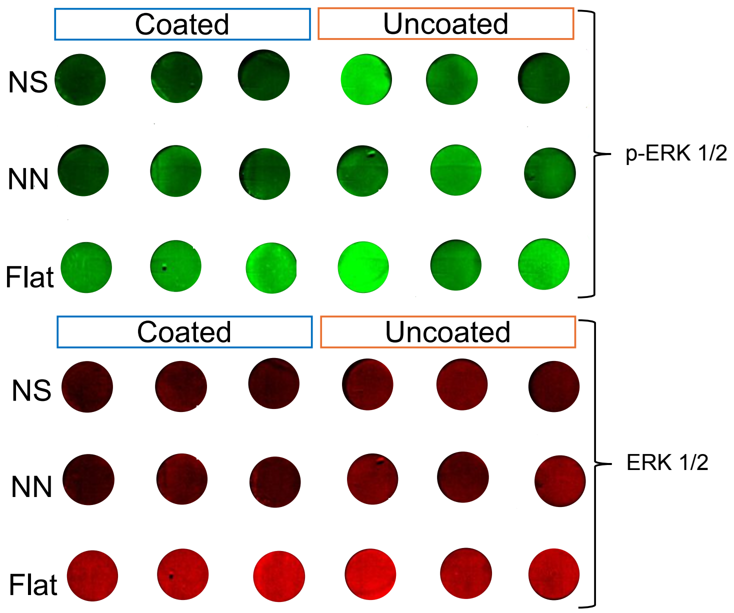

Figure S15. Representative images from in-cell western assay for p-ERK 1/2 ratio with ERK 1/2. hMSCs were incubated on flat, NS, or NN nanotopographies coated or uncoated, using co-culture media for 24 hours under a 5% CO_2 atmosphere at 37°C. Samples were fixed, stained for protein expression, read using a Licor Odyssey M, and analyzed using Empiria software.

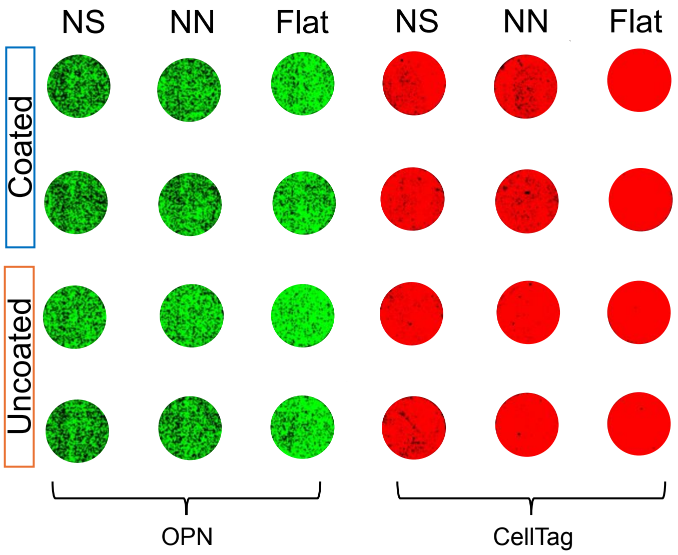

Figure S16. Representative images from in-cell western assay for osteopontin ratio with CellTag. hMSCs were incubated on flat, NS, or NN nanotopographies coated or uncoated, using co-culture media for 24 hours under a 5% CO_2 atmosphere at 37°C. Samples were fixed, stained for protein expression, read using a Licor Odyssey M, and analyzed using Empiria software.

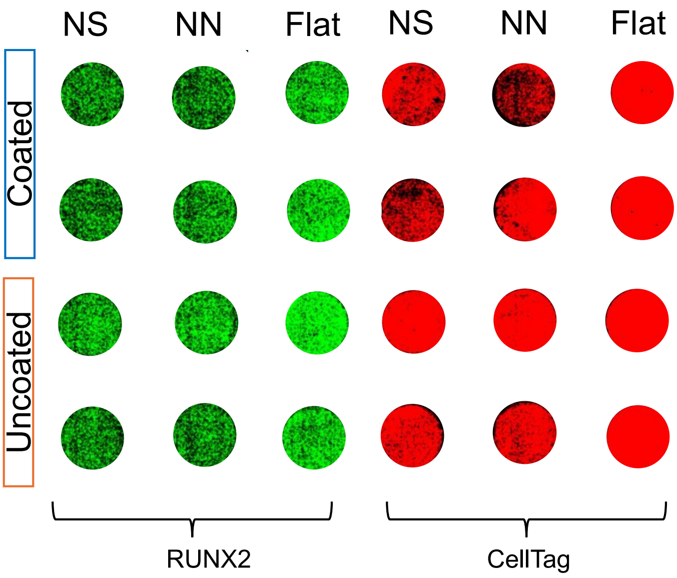

Figure S17. Representative images from in-cell western assay for RUNX2 ratio with CellTag. hMSCs were incubated on flat, NS, or NN nanotopographies coated or uncoated, using co-culture media for 24 hours under a 5% CO_2 atmosphere at 37°C. Samples were fixed, stained for protein expression, read using a Licor Odyssey M, and analyzed using Empiria software.

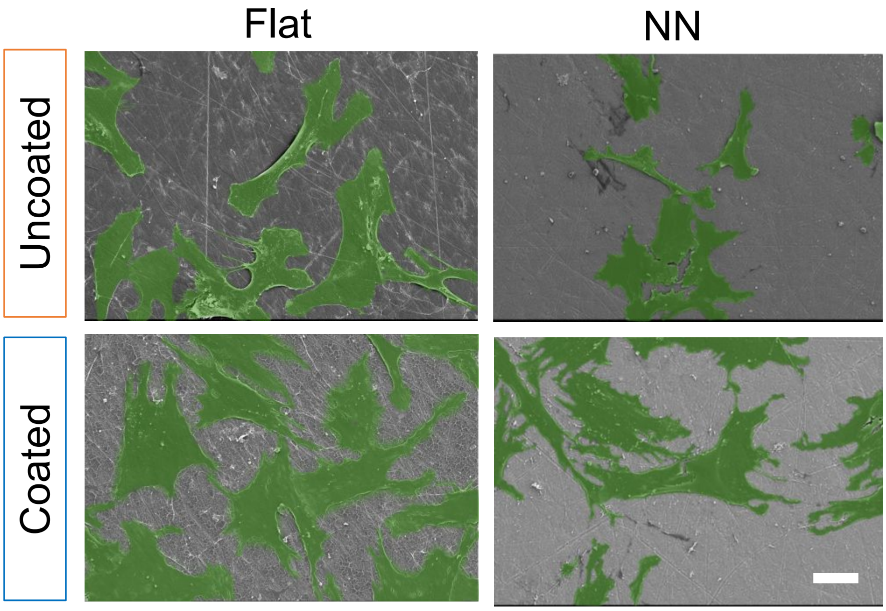

Figure S18. Representative images from scanning electron microscopy from co-culture incubation. Human mesenchymal stromal cells were seeded overnight on Flat and NN titanium surfaces, with or without coatings. The next day, the samples were treated with 10^3 CFU of *P. aeruginosa* for 24 hours under a 5% CO_2 atmosphere at 37°C. Samples were fixed, dehydrated, and coated for observation under scanning electron microscope JEOL IT100. Scale bar, 50 µm.

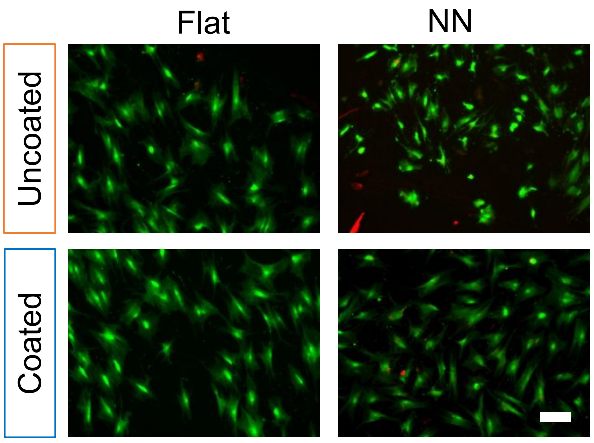

Figure S19. Representative images from live/ead assay from co-culture of human mesenchymal stromal cells (hMSCs) and *P. aeruginosa*. hMSCs were seeded overnight on Flat, NS and NN titanium surfaces, with or without coatings. The next day, the samples were treated with 10^3 CFU of *P. aeruginosa* for 24 hours under a 5% CO_2 atmosphere at 37°C. A Live/Dead assay was performed and the viable hMSCs were quantified and analyzed using Fiji software. Scale bar, 50 µm.

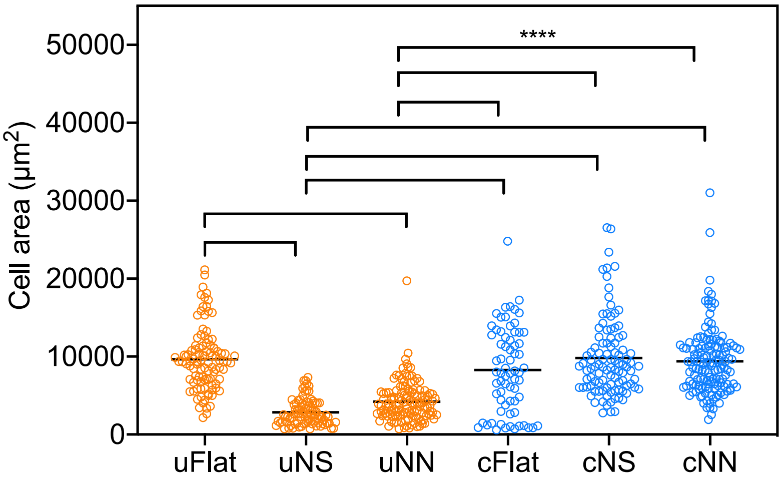

Figure S20. Cell area quantification from co-culture of human mesenchymal stromal cells (hMSCs) and *P. aeruginosa.* hMSCs were seeded overnight on Flat, NS and NN titanium surfaces, with or without coatings. The next day, the samples were treated with 10^3 CFU of *P. aeruginosa* for 24 hours under a 5% CO_2 atmosphere at 37°C. A live/dead assay was performed and the viable hMSCs were quantified and analyzed for area using Fiji software. Comparison of differences was tested using Kruskal-Wallis test with a p-value <0.0001 (****) considered highly significant. All data with brackets have significance of ****.

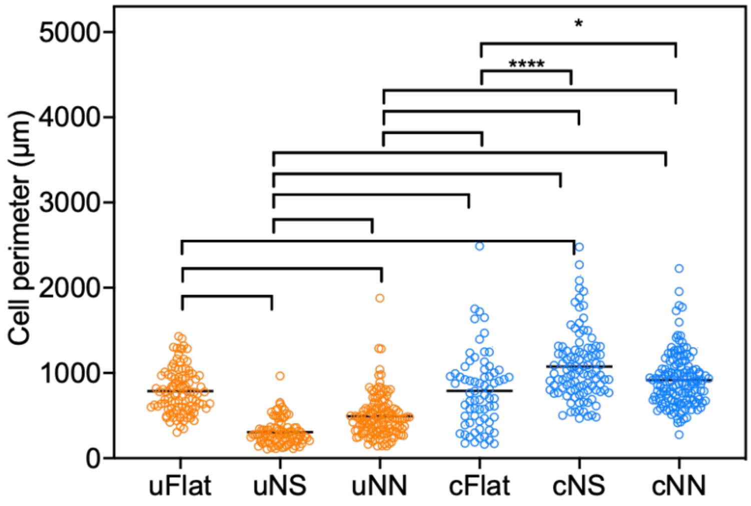

Figure S21. Cell perimeter quantification from co-culture of human mesenchymal stromal cells (hMSCs) and *P. aeruginosa*. hMSCs were seeded overnight on Flat, NS and NN titanium surfaces, with or without coatings. The next day, the samples were treated with 10^3 CFU of *P. aeruginosa* for 24 hours under a 5% CO_2 atmosphere at 37°C. A live/dead assay was performed and the viable hMSCs were quantified and analyzed for perimeter using Fiji software. Comparison of differences was tested using Kruskal-Wallis test with a p-value <0.05 (*) and <0.0001 (****) considered significant and highly significant, respectively. All data with brackets have a significance of **** except where * is indicated.

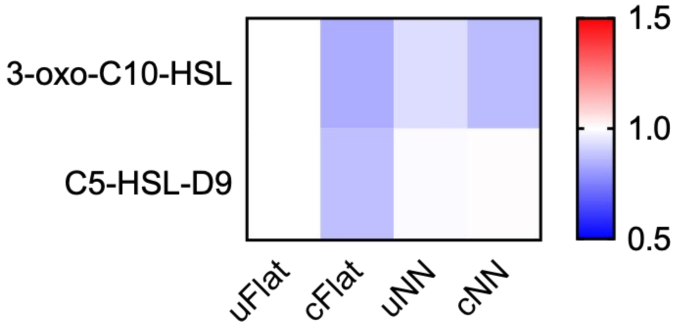

Figure S22. **Identified quorum sensing molecules in planktonic *P. aeruginosa* cells after co-culture with mesenchymal stromal cells (hMSCs).** hMSCs were seeded overnight on Flat, NS and NN titanium surfaces, with or without coatings. The next day, the samples were treated with 10^3 CFU of *P. aeruginosa* for 24 hours under a 5% CO_2 atmosphere at 37°C. Planktonic cells were recovered by pipetting and metabolites extracted using 1:3:1 water:ethanol:chloroform buffer, and analyzed by mass spectrometry. Mean values shown as heatmap, where red is upregulated, and blue is downregulated compared to uFlat surface. Comparison of differences was tested using Kruskal-Wallis test.

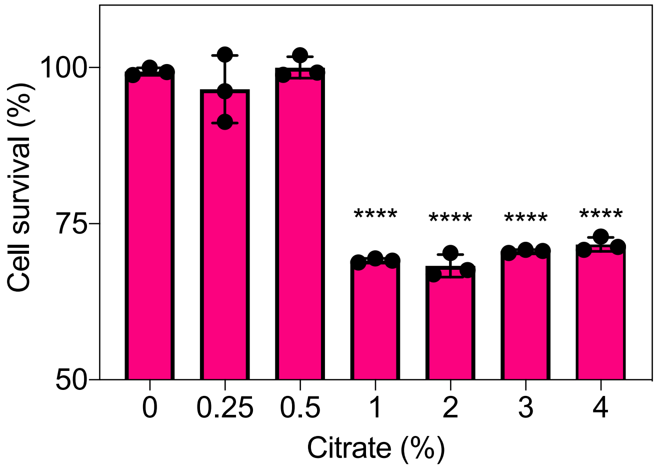

Figure S23. The effect of citrate on human mensenchymal stromal cells (hMSCs). hMSCs were seeded overnight and the next day, they were incubated in media containing incrementing concentrations of citrate 0, 0.25, 0.5, 1, 2, and 4 % weight/volume for 24 hours under a 5% CO2 atmosphere at 37 °C. An alamar blue assay was performed and the absorbance was measured at 570 nm. The samples were normalised to hMSC control without citrate. Mean values represented as bars with individual values and standard deviation. Comparison of differences was tested using Kruskal-Wallis test with a p-value <0.05 (*) and <0.0001 (****) considered significant and highly significant, respectively.

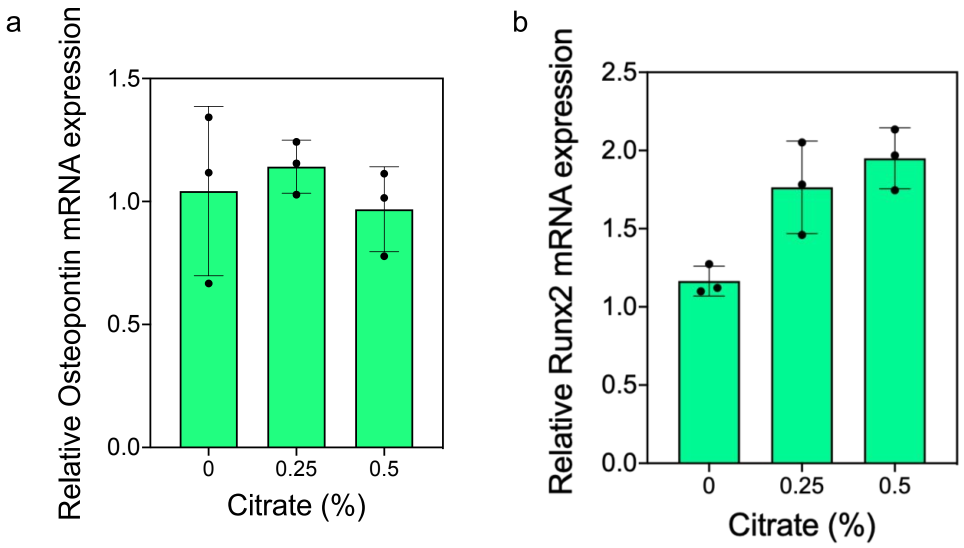

Figure S24. The effect of citrate on hMSC differentiation. hMSCs were incubated in osteogenic media containing concentrations of citrate 0, 0.25, and 0.5 % (w/v) for 14 days under a 5% CO_2 atmosphere at 37 °C. mRNA extraction was performed, and qPCR performed for **a** *osteopontin*, and **b** *Runx2*. The results were normalised to housekeeping genes *GAPDH* and *RPL13A*. Mean represented as bars with individual values and standard deviation. Comparison of differences was tested using Kruskal-Wallis test.

Table S1. List of QSMs present in *P. aeruginosa*, adapted from Ortori et al.[39]

Analyte	Precursor ion (m/z)	Product ion (m/z)	Declustering potential (V)	Collision energy (eV)
C4-HSL	172.1	102.1	26	15
C6-HSL	200.1	102.1	31	15
C8-HSL	228.1	102.1	26	15
C10-HSL	256.1	102.1	31	17
C12-HSL	284.1	102.1	31	17
C14-HSL	312.1	102.1	45	19
3-oxo-C4-HSL	186.1	102.1	26	15
3-oxo-C6-HSL	214.1	102.1	26	15
3-oxo-C8-HSL	242.1	102.1	26	17
3-oxo-C10-HSL	270.1	102.1	26	17
3-oxo-C12-HSL	298.1	102.1	30	19
3-oxo-C14-HSL	326.2	102.1	30	21
3-OH-C4-HSL	188.1	102.1	26	15
3-OH-C6-HSL	216.1	102.1	26	15
3-OH-C8-HSL	244.1	102.1	31	17
3-OH-C10-HSL	272.1	102.1	35	17
3-OH-C12-HSL	300.1	102.1	35	19
3-OH-C14-HSL	328.2	102.1	21	19
HHQ	244.1	159.1	31	20
NHQ	272.1	159.1	81	27
UHQ	300.1	159.1	81	27
HQNO	260.1	159.1	71	32
NQNO	288.1	159.1	96	20
PQS	260.1	175.1	71	22
C9-PQS	288.1	175.1	111	25
C11-PQS	316.1	175.1	116	35
C5-HSL-d9[a]	195.1	103.1	40	15
C10-TMA[b]	298.1	144.1	30	20
OR-3-oxo-C12-HSL[c]	316.2	298.1	28	12

[a]Internal standards
[b]Tetramic acid product of 3-oxo-C_{12}-HSL [c]Open-ring forms of 3-oxo-C_{12}-HSL

Table S2. List of intracellular QSMs screened in *P. aeruginosa* monoculture after incubation on coated and uncoated Flat, NS, and NN surfaces.

Analyte	Screened	Present in biofilm bacteria	Present in planktonic bacteria
C4-HSL	Yes	No	No
C6-HSL	Yes	Yes	Yes
C8-HSL	Yes	Yes	Yes
C10-HSL	Yes	Yes	Yes
C12-HSL	Yes	Yes	Yes
C14-HSL	Yes	Yes	Yes
3-oxo-C4-HSL	Yes	No	No
3-oxo-C6-HSL	Yes	No	No
3-oxo-C8-HSL	Yes	Yes	Yes
3-oxo-C10-HSL	Yes	Yes	Yes
3-oxo-C12-HSL	Yes	Yes	Yes
3-oxo-C14-HSL	Yes	Yes	Yes
3OH-C4-HSL	Yes	No	No
3OH-C6-HSL	Yes	No	No
3OH-C8-HSL	Yes	No	No
3OH-C10-HSL	Yes	No	No
3OH-C12-HSL	Yes	Yes	Yes
3OH-C14-HSL	Yes	No	No
HHQ	Yes	No	No
NHQ	Yes	No	No
UHQ	Yes	Yes	Yes
HQNO	Yes	No	No
NQNO	Yes	No	No
PQS	Yes	No	No
C9-PQS	Yes	No	No
C11-PQS	Yes	No	No
C5-HSL-D9	Yes	No	No
C10-TMA	No	No	No
OR-3-oxo-C12	No	No	No

Table S3. Possible functions of QSM in *P. aeruginosa* according to the literature.

Molecule	Possible function in P. aeruginosa
C6-HSL	Added exogenously increases the formation of biofilm.[1]
C8-HSL	Potential role in regulating efflux pumps.[2]
C10-HSL	Is present in QscR transcriptor factor.[3]
C12-HSL	Autoinducer of the Las system.[4]
C14-HSL	Possible implication on the QscR orphan quorum sensing regulator.[5]
3oxo-C8-HSL	Part of the LasR autoinduction pathway system for QSM biofilm formation.[6]
3oxo-C10-HSL	Part of the LasR autoinduction pathway system for QSM biofilm formation.[6]
3oxo-C12-HSL	Part of the LasR autoinduction pathway system for QSM biofilm formation.[6]
3oxo-C14-HSL	Part of the LasR autoinduction pathway system for QSM biofilm formation.[6]
3OH-C12-HSL	Part of the LasR autoinduction pathway system for QSM biofilm formation.[6]
UHQ	Part of the alkylquinolones, act as autoinducers, accumulating in the bacteria cell until a signal threshold is reached, activating a number of virulence-related genes. Furthermore, they also have activity against competitor bacteria.[7], [8]

Table S4. List of intracellular QSMs screened in *P. aeruginosa* after co-culture with hMSCs on coated and uncoated Flat or NN surfaces.

Analyte	Screened	Present in biofilm bacteria	Present in planktonic bacteria
C4-HSL	Yes	No	No
C6-HSL	Yes	No	No
C8-HSL	Yes	No	No
C10-HSL	Yes	Yes	No
C12-HSL	Yes	Yes	No
C14-HSL	Yes	Yes	No
3-oxo-C4-HSL	Yes	No	No
3-oxo-C6-HSL	Yes	No	No
3-oxo-C8-HSL	Yes	Yes	No
3-oxo-C10-HSL	Yes	Yes	Yes
3-oxo-C12-HSL	Yes	Yes	No
3-oxo-C14-HSL	Yes	Yes	No
3OH-C4-HSL	Yes	No	No
3OH-C6-HSL	Yes	No	No
3OH-C8-HSL	Yes	No	No
3OH-C10-HSL	Yes	Yes	No
3OH-C12-HSL	Yes	Yes	No
3OH-C14-HSL	Yes	No	No
HHQ	Yes	No	No
NHQ	Yes	No	No
UHQ	Yes	No	No
HQNO	Yes	No	No
NQNO	Yes	No	No
PQS	Yes	No	No
C9-PQS	Yes	No	No
C11-PQS	Yes	No	No
C5-HSL-D9	Yes	Yes	Yes
C10-TMA	Yes	No	No
OR-3-oxo-C12	Yes	Yes	No

Table S5. The top 12 changes in the intracellular metabolome map family of human mesenchymal stromal cells (hMSCs). hMSCs were co-cultured with *P. aeruginosa* for 24 h, and the metabolites in the hMSCs were then extracted and analyzed using Metaboanalyst.

**Metabolite**	**Map**	**Pathway**
Octadecanoic acid	Lipid Metabolism	Fatty acid biosynthesis<br>Biosynthesis of unsaturated fatty acids
Hexadecanoic acid	Lipid Metabolism	Fatty acid biosynthesis<br>Fatty acid elongation in mitochondria<br>Fatty acid metabolism<br>Biosynthesis of unsaturated fatty acids
[FA (17:0)] heptadecanoic acid	Lipids: Fatty Acyls	Fatty Acids and Conjugates
3-Hydroxyestra-1,3,5(10),6-tetraen-17-one	Lipids: Sterol lipids	Steroids
[Fv] Dihydrochalcone	Lipids: Polyketides	Flavonoids
4-Hydroxy-4-methylglutamate	Carbohydrate Metabolism	C5-Branched dibasic acid metabolism
2-Acetolactate	Carbohydrate Metabolism	Butanoate metabolism<br>Pantothenate and CoA biosynthesis
4-Methylene-L-glutamine	Carbohydrate Metabolism	C5-Branched dibasic acid metabolism
Thr-Ala	Peptide(di-)	Polar peptide
Phosphocreatine	Amino Acid Metabolism	Arginine and proline metabolism
Glutathione	Amino Acid Metabolism	Glutamate metabolism<br>Cysteine metabolism<br>Glutathione metabolism
Citrate	Carbohydrate Metabolism	Citrate cycle (TCA cycle)<br>Glutamate metabolism<br>Alanine and aspartate metabolism<br>Glyoxylate and dicarboxylate metabolism<br>Reductive carboxylate cycle (CO2 fixation)

Table S6. The top 12 changes in the secreted metabolome map family of human mesenchymal stromal cells (hMSCs). hMSCs were co-cultured with *P. aeruginosa* for 24 h and the metabolites of the hMSC secretome were then extracted and analyzed using Metaboanalyst.

**Metabolite**	**Map**	**Pathway**
2-Oxoglutamarate	Carbohydrate Metabolism	Citrate cycle (TCA cycle)<br>Ascorbate and aldarate metabolism<br>Glutamate metabolism<br>Alanine and aspartate metabolism<br>Lysine biosynthesis<br>Histidine metabolism<br>D-Glutamine and D-glutamate metabolism<br>Glyoxylate and dicarboxylate metabolism
5-Hydroxyindoleacetate	Amino Acid Metabolism	Tryptophan metabolism
Val-Ser	Peptide(di-)	Hydrophobic peptide
O-methylandrocymbine	Biosynthesis of Secondary Metabolites	colchicine biosynthesis
Asp-Pro-Arg	Peptide(tri-)	Basic peptide
N4-Acetylaminobutanal	Amino Acid Metabolism	Arginine and proline metabolism
5-Hydroxy-L-tryptophan	Amino Acid Metabolism	Tryptophan metabolism
5-Hydroxy-L-tryptophan	Amino Acid Metabolism	Tryptophan metabolism
3,4-Dihydroxy-L-phenylalanine	Amino Acid Metabolism	Tyrosine metabolism
Trp-Ser-Tyr	Peptide(tri-)	Hydrophobic peptide
(S)-1-Pyrroline-5-carboxylate	Amino Acid Metabolism	Glutamate metabolism<br>Arginine and proline metabolism
3-Methyl-2-oxobutanoic acid	Amino Acid Metabolism	Valine, leucine, and isoleucine degradation<br>Valine, leucine, and isoleucine biosynthesis<br>Pantothenate and CoA biosynthesis

Supplementary Methods

Energy dispersive X-ray spectroscopy

pPEA coated Flat, NS, NN, and their uncoated counterparts were mounted in a stub using carbon tape, and a small amount of aluminium tape was added to ensure conductivity on the sample. The samples were then loaded into the chamber at high vacuum in a Zeiss Gemini SEM microscope. Aperture at 60 µm, acceleration at 15 kV and a working distance of 8 mm. The data was taken from six different points on each sample and plotted as weight percentage of the total using Oxford Aztec software.

P. aeruginosa CFU and antibiotic titration experiments

The strain *P. aeruginosa* ATCC 27853 was used and 10 µL of a glycerol stock was used to inoculate 10 mL of Dulbecco's modified eagle medium (DMEM) without any supplements. The next day, the bacterial suspension was diluted to optical density (OD) 600 0.1 in DMEM without supplements and used at its log phase at OD600=0.3, equivalent to 10^8 colony forming units (CFU). This was further diluted to 10^6, 10^4, 10^3, 10^2 CFU mL^{-1} for all experiments in co-culture media (DMEM supplemented with 1% foetal bovine serum (FBS), 1% Eagle's minimum essential medium non-essential amino acid solution (MEM NEAA, Gibco), 1% L-glutamine, and 1% sodium pyruvate), and final concentrations of penicillin/streptomycin (Sigma) 1%, 0.5%, 0.4%, 0.3%, and 0.2%. A 0% concentration was used to monitor the normal growth of *P. aeruginosa*. 1 mL of each condition was seeded in a 24 well plate and incubated overnight at 37°C. (Figure S1a). The next day, a BacTiter Glo (Promega, G8231) assay was performed. Briefly, 100 µL of the grown *P. aeruginosa* were placed in an opaque white 96-well plate with 100 µL of BacTiter Glo solution. The luminescence intensity was measured using CLARIOstar (BMG LABTECH, Germany) plate reader. From the same 24-well plate, another 100 µL was taken and further diluted 10^{-2}, 10^{-3}, 10^{-4}, 10^{-5}, 10^{-6}, 10^{-7} and a 20 µL drop was placed onto an agar plate and incubated overnight to enable determination of viable counts.

Bacterial metabolic activity

BacTiter-Glo (G819A, Promega) was used to measure the adenosine triphosphate (ATP) metabolic activity levels of *P. aeruginosa* as an indication of viability. *P. aeruginosa* was cultured as described in previous section. Bacterial suspension at 10^3 CFU in co-culture media was added to the Ti Flat, NS, and NN coated or uncoated,

and incubated in a humidified incubator at 37 ºC under a 5% CO_2 atmosphere. The next day, 100 µL of the bacterial suspension was mixed with 100 µL of BacTier-Glo in a white 96 well plate and incubated for 5 minutes in the dark. The luminescence intensity was measured using a CLARIOstar (BMG LABTECH, Germany) plate reader.

Human mesenchymal stromal cell culture

Stro-1+ selected human primary mesenchymal stromal cells (hMSCs) were provided by Southampton General Hospital (ASK LREC194/99/1 and 18/NW/0231 and 210/01). hMSCs were cultured in Dulbecco's modified essential media (DMEM) supplemented with 10% foetal bovine serum (FBS), 6.74 $U{\cdot}mL^{-1}$ penicillin/streptomycin, 1% Eagle's minimum essential medium non-essential amino acid solution (MEM NEAA, Gibco), 1% L-glutamine, and 1% sodium pyruvate. The hMSCs were cultured in a humidified incubator at 37 ºC under a 5% CO_2 atmosphere. Cells were expanded in monolayer using T75 flasks using 10 to 12 mL of media with changes every other day.

hMSC area and perimeter

From the hMSC viability experiment, the obtained images were converted to 8 bit and a threshold was applied before the living cells (green) were quantified, and their area and perimeter measured using the Analyze Particles function.

hMSC viability in citrate

Citrate was prepared in different concentrations 5, 4, 3, 2, 1, 0.5, and 0.5% w/v in DMEM co-culture media. 10,000 hMSCs were seeded in a 24 well plate, and the next day the media was changed containing different concentrations of citrate. An alamar blue assay was performed (Biorad) following the instructions of the manufacturer. After 4 hours, 100 µL of the media in triplicate were used for absorbance measurements at 570 nm.

hMSC differentiation in citrate

hMSCs on a 24 well plate were cultured for 14 days in co-culture media (containing 1% FBS and 0.3% penicillin/streptomycin) and 0.25% citrate. The content of wells was pooled to have sufficient RNA. The RNA was isolated using a RNeasy kit (Qiagen) following the instructions of the manufacturer. cDNA isolation and qPCR quantification were carried out as previously described.

Crystal violet staining

Citrate was prepared at 0.5% or 5.0% (w/v) in DMEM co-culture media. *P. aeruginosa* (10^3 CFU) was seeded in a 24 well plate in media containing citrate with or without penicillin/streptomycin. The next day, the media was removed and the biofilm layer was fixed with 100% ice cold methanol for 15 minutes at –20°C. The biofilm was rinsed with PBS twice and then 1 mL of 0.5% (w/v) of crystal violet in 20% methanol was added to each well. After 15 minutes, the crystal violet solution was removed and the wells were washed four times with dH_2O and left to dry. The absorbance of the stained biofilm was read using an Odyssey M Licor at 700 nm.

Bibliography

(1) Feng, Q.; Luo, L.; Chen, X.; Zhang, K.; Fang, F.; Xue, Z.; Li, C.; Cao, J.; Luo, J. Facilitating Biofilm Formation of Pseudomonas Aeruginosa via Exogenous N-Acy-L-Homoserine Lactones Stimulation: Regulation on the Bacterial Motility, Adhesive Ability and Metabolic Activity. *Bioresour Technol* **2021**, *341*, 125727. https://doi.org/10.1016/J.BIORTECH.2021.125727.

(2) Yawata, Y.; Maseda, H.; Okabe, S.; Ito, A.; Sawada, I.; Kurashima, H.; Uchiyama, H.; Nomura, N. The Response of Pseudomonas Aeruginosa PAO1 Efflux Pump-Defective Mutants to N-Octanoyl-L-Homoserine Lactone. *Microbes Environ* **2009**, *24* (4), 338–342. https://doi.org/10.1264/JSME2.ME09130.

(3) Ding, F.; Oinuma, K. I.; Smalley, N. E.; Schaefer, A. L.; Hamwy, O.; Greenberg, E. P.; Dandekar, A. A. The Pseudomonas Aeruginosa Orphan Quorum Sensing Signal Receptor QscR Regulates Global Quorum Sensing Gene Expression by Activating a Single Linked Operon. *mBio* **2018**, *9* (4). https://doi.org/10.1128/MBIO.01274-18/SUPPL_FILE/MBO004184035ST2.DOCX.

(4) Shahbandeh, M.; Moosazadeh Moghaddam, M.; Golmohammadi, R.; Mirnejad, R. The Antimicrobial Effect of Quorum Sensing Autoinducers of Pseudomonas Aeruginosa, C12-HSL and C4-HSL, against MDR Staphylococcus Aureus Isolates. *Comp Immunol Microbiol Infect Dis* **2022**, *81*, 101747. https://doi.org/10.1016/J.CIMID.2022.101747.

(5) Chugani, S.; Greenberg, E. P. An Evolving Perspective on the Pseudomonas Aeruginosa Orphan Quorum Sensing Regulator QscR. *Front Cell Infect Microbiol* **2014**, *4* (OCT), 116921. https://doi.org/10.3389/FCIMB.2014.00152/BIBTEX.

(6) Liu, Y. C.; Chan, K. G.; Chang, C. Y. Modulation of Host Biology by Pseudomonas Aeruginosa Quorum Sensing Signal Molecules: Messengers or Traitors. *Front Microbiol* **2015**, *6* (NOV), 168355. https://doi.org/10.3389/FMICB.2015.01226/BIBTEX.
(7) Heeb, S.; Fletcher, M. P.; Chhabra, S. R.; Diggle, S. P.; Williams, P.; Cámara, M. Quinolones: From Antibiotics to Autoinducers. *FEMS Microbiol Rev* **2011**, *35* (2), 247. https://doi.org/10.1111/J.1574-6976.2010.00247.X.
(8) Saalim, M.; Villegas-Moreno, J.; Clark, B. R. Bacterial Alkyl-4-Quinolones: Discovery, Structural Diversity and Biological Properties. *Molecules* **2020**, *25* (23). https://doi.org/10.3390/MOLECULES25235689.
